# Production of novel pladienolide analogues through native expression of a pathway-specific activator†

**DOI:** 10.1039/d0sc01928c

**Published:** 2020-07-17

**Authors:** Thomas J. Booth, John A. Kalaitzis, Daniel Vuong, Andrew Crombie, Ernest Lacey, Andrew M. Piggott, Barrie Wilkinson

**Affiliations:** Department of Molecular Microbiology, John Innes Centre Norwich Research Park Norwich NR4 7UH UK barrie.wilkinson@jic.ac.uk; Department of Molecular Sciences, Macquarie University NSW 2109 Australia andrew.piggott@mq.edu.au; Microbial Screening Technologies Smithfield NSW 2164 Australia elacey@microbialscreening.com

## Abstract

Aberrant splicing of pre-mRNA is implicated in many human genetic disorders. Small molecules that target the spliceosome are important leads as therapeutics and research tools, and one compound of significant interest is the polyketide natural product pladienolide B. Here, we describe the reactivation of quiescent pladienolide B production in the domesticated lab strain *Streptomyces platensis* AS6200 by overexpression of the pathway-specific activator PldR. The resulting dysregulation of the biosynthetic genes led to the accumulation and isolation of five additional intermediate or shunt metabolites of pladienolide B biosynthesis, including three previously unreported congeners. These compounds likely comprise the entire pladienolide biosynthetic pathway and demonstrate the link between polyketide tailoring reactions and bioactivity, particularly the importance of the 18,19-epoxide. Each congener demonstrated specific inhibitory activity against mammalian cell lines, with successive modifications leading to increased activity (IC_50_: 8 mM to 5 μM).

## Abstract 2

Reactivation of quiescent polyketide production in a domesticated lab strain.

## Graphical abstract

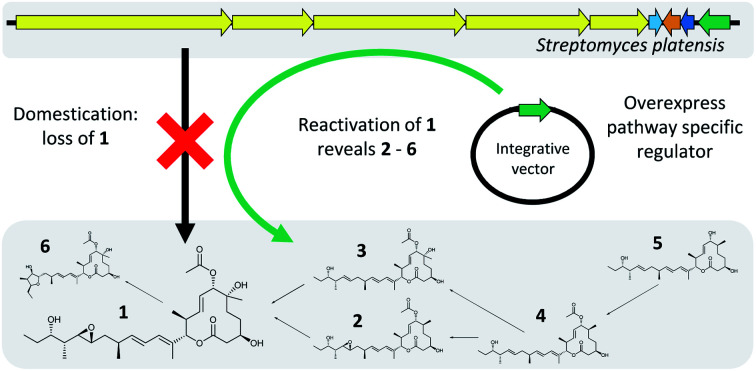

Pre-mRNA splicing is an important mechanism in eukaryotic gene expression whereby non-coding introns are removed to produce mature mRNA. Splicing is controlled by the spliceosome, a large protein-RNA complex assembled from multiple ribonucleoprotein particles. Given that over 95% of the genes encoded by the human genome contain introns, correct assembly of the spliceosome complex and the precise coordination of splicing is vital for correct cellular function.^1^ Aberrant splicing of pre-mRNA is implicated in many human genetic disorders including retinal degeneration, muscular and neurological diseases, and cancer,^2,3^ therefore small molecules that can modulate splicing are important as potential pharmaceutical agents and as research tools for probing the function of the spliceosome. Three families of bacterial polyketide natural products are known to impair the assembly of the spliceosome: spliceostatins,^4,5^ herboxidienes^6,7^ and pladienolides^8–10^ (Fig. 1a). All three classes function through a common pharmacophore consisting of a conjugated diene. By binding within a tunnel between two components of the spliceosome, SF3b and PHF5A, the correct assembly of the spliceosome is prevented, ultimately resulting in cell cycle arrest.^11,12^

**Fig. 1:**
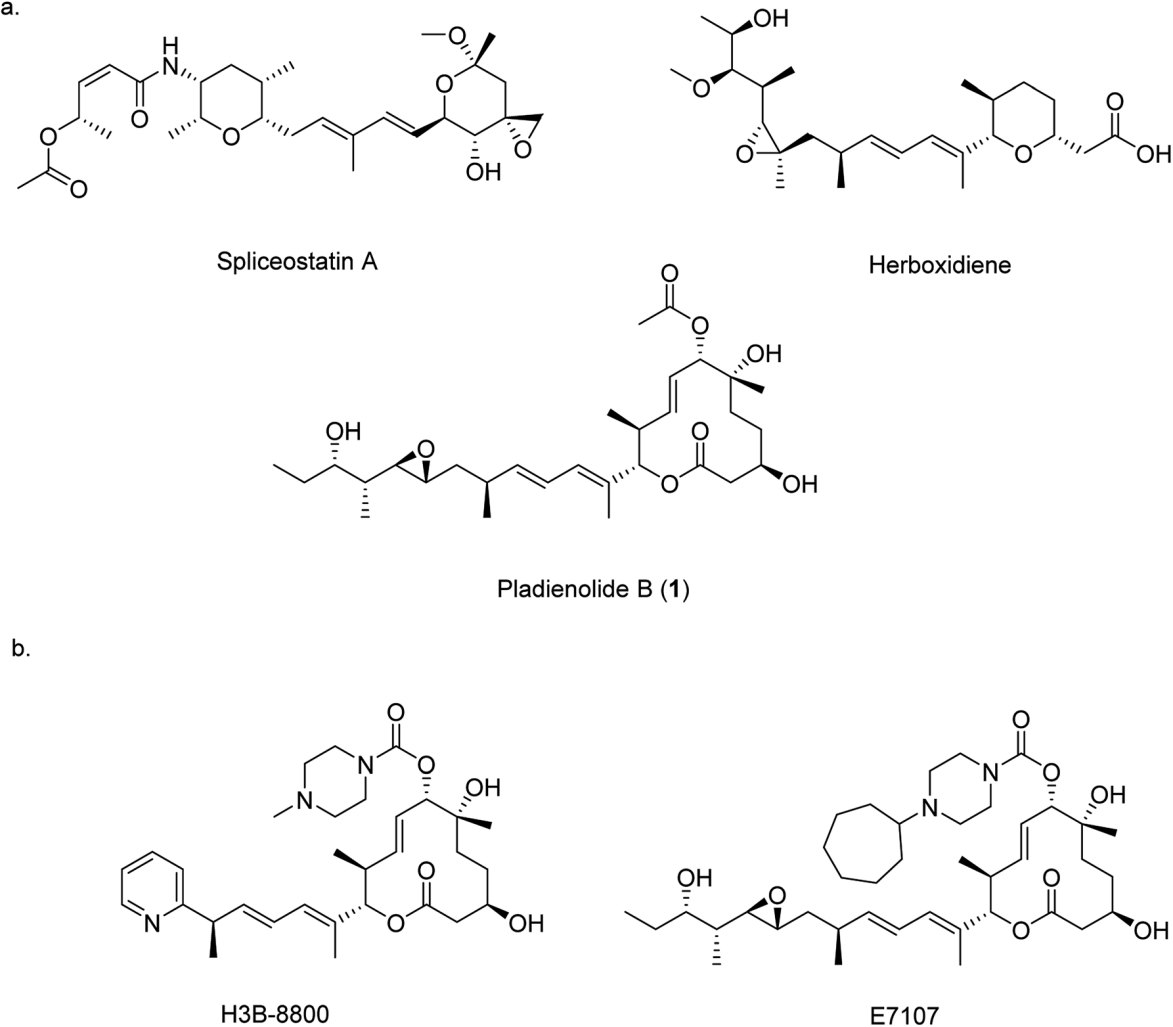
Chemical structures of natural product splicing modulators: (a) spliceostatin A from *Burkholderia* sp. No. 2663, herboxidiene from *Streptomyces chromofuscus* ATCC 49982, and pladienolide B (**1**) from *Streptomyces platensis* Mer-11107 and; (b) synthetic analogues of **1**.

Given their therapeutic potential, there has been significant interest in the generation of new spliceosome inhibiting analogues. For example, several analogues of herboxidiene have been generated through the modification of the carboxylic acid. Analogues of pladienolide B (**1**) have also been generated through derivatisation of the *O*-acyl group and modification of the polyketide tail.^13–16^ These analogues have enjoyed some clinical success, and two analogues (E7107 and H3B-8800 (Fig. 1b)) have entered into clinical trials for the treatment of solid tumours.^14–17^

We have studied two strains of *Streptomyces platensis* that are known producers of **1**, AS6200 (a derivative of ATCC 23948) and MA5455 (identified from the Microbial Screening Technologies culture collection). During domestication for large scale culture, these strains lost the ability to produce **1**. Domestication is a process of artificial selection, whereby an organism is adapted to an anthropogenic niche. Current literature has focused almost exclusively on the domestication of industrial microorganisms,^18^ however, given the nature of laboratory culture (*e.g.* the requirement of monoculture, the use of homogenous media, unconscious selection for uniform/rapid growth *etc.*) and the rapidly evolving nature of microbial communities, domestication is also an inevitable process in a research environment. Although the phenotypic changes associated with domestication are effectively limitless, they commonly encompass changes to reproductive pathways (*e.g.* loss of sexual reproduction or the ability to sporulate),^19^ utilisation of metabolic substrates (*e.g.* sugars and amino acids),^20^ or the production of specialised metabolites.^21,22^ Such phenotypes arise either through pseudogenisation, or changes in regulatory pathways.^20,23^ Although some traits are desirable, strategies to mitigate unwanted consequences of domestication merit further investigation.

Pladienolide biosynthesis is encoded by the *pld* biosynthetic gene cluster (BGC), described in 2008 (Fig. 2).^24^ It comprises eight genes which putatively encode four polyketide synthases (PKSs) totalling 10 modules (PldAI-IV), three post-PKS tailoring enzymes (PldBCD), and a transcriptional regulator (PldR). The PKS PldAI-IV consists of KS_Q_ starter module and 11 extension modules responsible for the biosynthesis of the undecaketide scaffold and subsequent lactonisation. A *pldB* disrupted strain was only capable of producing 6-deoxypladienolide B (**2**), establishing the role of PldB as the C6-hydroxylase. The role of PldD was inferred as a C18/19 epoxidase due to the similarity between *pldD* and *monC1* and *nanO* (monensin and nanchangmycin epoxidases respectively).^25,26^ PldC is implicated as a 7-*O*-acetyltransferase due to its homology with Rif-Orf20.^27^ PldR is a member of the large ATP-binding regulators of the LuxR family (LALs). LALs are characterised by their domain architecture, possessing an N-terminal ATP-binding domain and a C-terminal helix-turn-helix domain.^28^ Although LALs have been reported as repressors and pleiotropic activators, the majority act as pathway-specific activators.^29–33^ Given the simple architecture of the *pld* BGC, and the fact that LuxR family regulators generally act in a positive manner, we rationalised that overexpression of *pldR* should restore pladienolide production in domesticated *S. platensis* strains which had lost the ability to produce **1**.

**Fig. 2:**
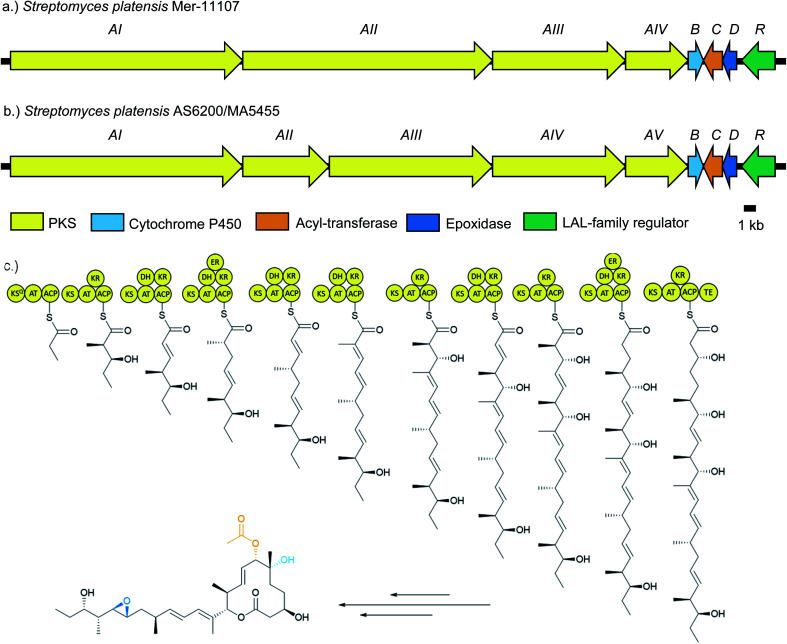
Comparison of the pladienolide (pld) biosynthetic gene clusters of (a) *S. platensis* Mer-11107 and (b) *S. platensis* AS6200 and *S. platensis* MA5455 and (c) pladienolide B (**1**) biosynthetic pathway including polyketide synthesis and tailoring steps.

Genomic DNA of two pladienolide-producing *S. platensis* strains (AS6200 and MA5455) was sequenced using the Pacific Biosciences (PacBio) RSII platform^34^ and the genomes assembled with HGAP 3.0.^35^*S. platensis* AS6200 was assembled in four contigs (4.65 Mb, 3.98 Mb, 309 kb and 22 kb) and *S. platensis* MA5455 in two contigs (8.61 Mb and 297 kb) (Table S3†), presumably representing the chromosome and a megaplasmid. Overall, the two genome sequences were highly similar (99.4% pairwise nucleotide identity, Table S4†) and on this basis only the genome sequence for *S. platensis* AS6200 was deposited (GenBank: MN974405). Unsurprising, given their overall sequence similarity, both genomes contain the same array of BGCs and analysis using antiSMASH v 4.0 ^36^ identified 35 putative BGCs on the chromosome and a single BGC encoding a putative aryl-polyene on the linear plasmid. The pladienolide BGCs from both strains were easily identified due to the high similarity (>95% nucleotide similarity) with the published *pld* BGG.^24^ Overall, the architectures of all three *pld* BGCs are very similar and the sequences show very high similarity overall (Fig. 2 and Table S4†). The only notable difference is that the PKS is encoded by five genes in AS6200 and MA5455 as opposed to four in the previously reported BGC.

We rationalised that overexpression of the LAL *pldR* would be sufficient to re-establish pladienolide biosynthesis *in vivo*. To constitutively express PldR *in vivo*, *pldR* was cloned into the *Nde*I and *Hind*III sites of the integrative vector pGP9 ^37^ under the control of the *P*_act_ promoter and its cognate activator ActII-ORF4. The resulting plasmid pTJB1 was introduced into *S. platensis* AS6200 and MA5455 by conjugation; strains were also transformed with the empty vector (pGP9) to provide negative controls. Solvent extracts of apramycin-resistant ex-conjugants grown in a variety of solid and liquid media were assayed using LC(UV)MS to detect the presence of **1** (compared to an authentic standard). Most samples showed little variation between the overexpression strains and negative controls. However, extracts from AS6200 ex-conjugants containing pTJB1 (AS6242) cultured in media SM18, contained a peak with elution time and UV/MS characteristics corresponding to **1** ([M + H]^+^*m*/*z* 537.3428; calculated for C_30_H_49_O_8_^+^ 537.3432; *Δ* = −0.9 ppm).

Production of **1** was scaled up into 4 × 2 L fermentations and during this process, an additional five analogues of **1** were identified based on a common chromophore (Fig. S3†). Cultures of AS6242 were fermented for 14 days on SM18 media containing 1% C_18_ resin. Pelleted mycelia and resin were extracted with methanol (2 L) and partitioned with ethyl acetate (2 L) prior to further fractionation through isocratic preparative C_18_ HPLC (45% CH_3_CN/H_2_O) to yield **1** (31.9 mg) and **6** (2.1 mg). Further fractionation of the non-polar fraction *via* LH-20 chromatography and isocratic preparative C_18_ HPLC yielded **2** (3.0 mg), **3** (6.2 mg), **4** (6.4 mg) and **5** (3.9 mg) (Fig. S4†). The structures of **2–6** (Fig. 3) were then elucidated by detailed spectroscopic analysis (ESI†).

**Fig. 3:**
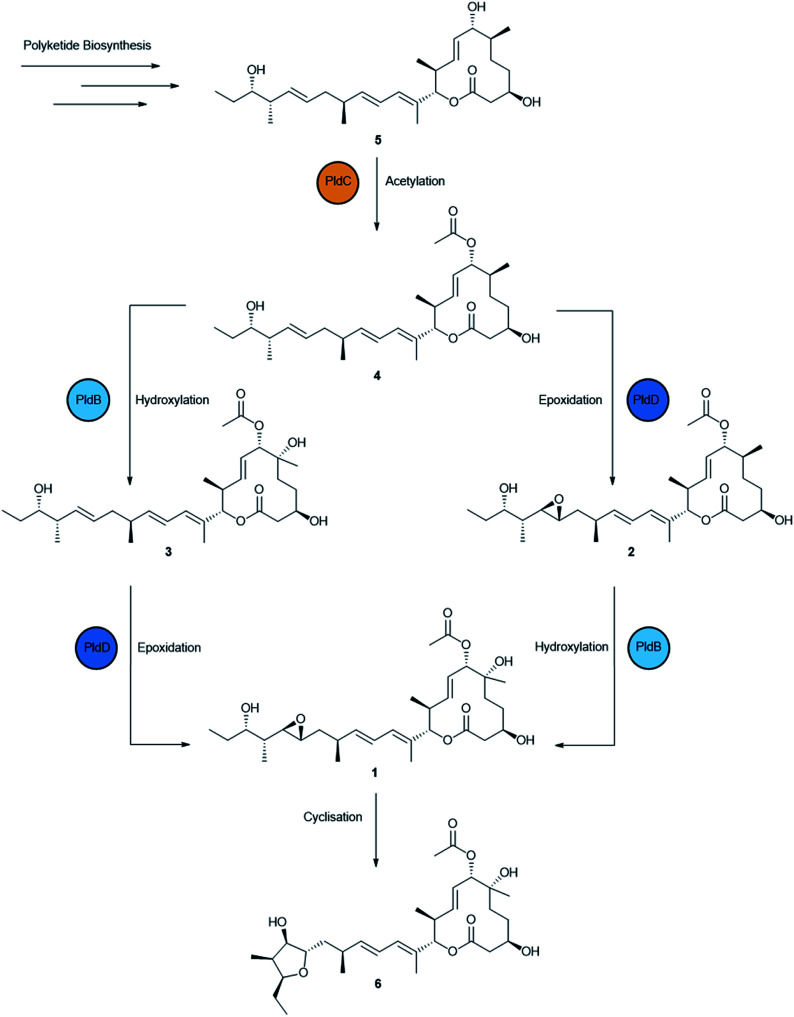
Biosynthesis of pladienolide B (**1**) and congeners (**2–6**) produced by *S. platensis* AS6242 identified in this study. The polyketide product of PldAI-V is acetylated by PldC followed by hydroxylation/epoxidation by PldB/PldD to produce **1**. Spontaneous intramolecular cyclisation of **1** yields **6**.

Compounds **2–5** are predicted to be pathway intermediates, presumably arising as a result of perturbing the regulation and stoichiometry of the biosynthetic machinery. Compound **2** (6-deoxypladenolide B) has been described previously as the only product of *pldB* disruption mutant consistent with this transformation occurring late in the biosynthetic pathway.^24^ Compound **3** is a 18,19-desepoxy derivative of **1** that was also described previously when the *pld* BGC and *pldR* were heterologously expressed in *Streptomyces avermitilis*.^38^ Compounds **4** and **5** are novel 18,19-desepoxy derivatives lacking the 6-hydroxy group, or the 6-hydroxy and the 7-*O*-acetyl groups, respectively. Compound **6** is unlikely to be a pathway intermediate and is more likely a shunt metabolite that results from spontaneous intramolecular cyclisation due to attack of the 21-hydroxy group on the 18,19-epoxide to form the tetrahydrofuranol.

To ascertain the bioactivity of all compounds the inhibitory concentrations of **1–6** were measured against bacteria (*Bacillus subtilis* ATCC 6633 and *Staphylococcus aureus* ATCC 25923), yeasts (*Candida albicans* ATCC 10231 and *Saccharomyces cerevisiae* ATCC 9763), a protozoan (*Tritrichomonas foetus*), and mammalian cell lines (NS-1 mouse myeloma ATCC TIB-18 and NFF human neonatal foreskin fibroblast cells ATCC PCS-201). Compounds **1–6** all demonstrated specific inhibitory activity against mammalian cultures with none of the compounds tested demonstrating activity against bacteria, fungi or protozoa (Table 1). Given this selectivity, the IC_50_ values for each compound **1–5***versus* NS-1, NFF and DU145 human prostate cancer cell ATCC HTB-81 were ascertained by a resazurin-based assay (Table 2). These data highlight the importance of post-PKS modifications for pladienolide activity, demonstrating how successive modifications improve activity. The loss of the 6-hydroxy group resulted in a slight loss of activity with **2** demonstrating activities ranging from those comparable with **1** up to around 8-fold less effective depending on the cell line. Loss of the 18,19-epoxide had a much greater effect on activity, as **3** recorded IC_50_ values up to twenty-five times greater than those of **1** and showed consistently lower inhibitory activity than **2** across all cell lines. Crystal structures of **1** bound to SF3B1 show that the weak polar interactions between 18,19-epoxide and V1110 play an important role in the orientation of the aliphatic tail,^12^ hence the lower activity of **3**. Unsurprisingly, **4** lacking both the 6-hydroxy group and the 18,19-epoxide shows lower activity against DU145 than both **2** and **3**. Furthermore, **5**, lacking all three tailoring modifications, exhibits the highest MIC/IC_50_ values demonstrating activity >600 times lower than **1** against NFF. Importantly, compounds **1–5** were less potent against NFF fibroblasts compared to the two tumour cell lines indicating selectivity against cancer cell lines.

**Table 1:** Minimum inhibitory concentrations (MICs) of pladienolide B (**1**) and its congeners (**2–6**) against NS-1 mouse myeloma ATCC TIB-18 and NFF human neonatal foreskin fibroblast cells ATCC PCS-201

Compound	MIC (μg mL^−1^)	
	NS-1	NFF
**1**	0.001	0.013
**2**	0.001	0.04
**3**	0.05	0.6
**4**	0.1	0.4
**5**	0.63	10
**6**	0.08	1.25

**Table 2:** IC_50_ values of pladienolide B (**1**) and its analogues (**2–5**) against mammalian cell lines: (NS-1 mouse myeloma ATCC TIB-18, DU145 human prostate cancer cell ATCC HTB-81 and NFF human neonatal foreskin fibroblast cells ATCC PCS-201). ND denotes no data

Compound	IC_50_ (μM) ± 95% confidence interval		
	NS-1	DU145	NFF
**1**	5.6 ± 0.6	7.5 ± 3.5	13.0 ± 4.1
**2**	40.3 ± 3.7	9.6 ± 5.8	27 ± 14
**3**	>770	96 ± 27	319 ± 61
**4**	>790	264 ± 79	>790
**5**	ND	ND	8100 ± 1800

The overexpression of pathway-specific activators is a well-established method for the discovery of new natural products and the improvements of yields.^29,31,39,40^ Here, we have shown that native overexpression of the pathway-specific activator *pldR* is sufficient to reactivate the production of **1** previously lost during the domestication process. In addition, upregulation of the pathway resulted in the accumulation of pladienolide congeners **2–6**. Interestingly, compounds **2–5** are putative pathway intermediates, including two previously undescribed compounds (**4** and **5**), and likely represent the entire post-PKS pladienolide biosynthetic pathway. This provides new insight into the biosynthesis of **1**. The first step in the pathway is most likely *O*-acetylation of the polyketide product by PldC. This appears to be a critical step in the biosynthetic pathway as we did not observe any desacetyl products with further modification. It is possible therefore that *O*-acetylation is required for efficient recognition of pladienolide by downstream enzymes. Secondly, PldB and PldD act independently to install the 6-hydroxy group and the 18,19-epoxide to produce mature **1**. Interestingly, this is a different complement of congeners than described by Sakai *et al.*,^8^ (pladienolide A and pladienolides C-G). Presumably, overexpression of PldR perturbs the stoichiometry of pathway enzymes in such a way that the downstream tailoring enzymes, PldBCD, are unable to turnover their substrates at a sufficient rate to ensure complete conversion of the nascent polyketide **5** to the final product **1**. Whatever the reason, this approach has implications for exploring the chemical diversity of any BGC, adding a previously unrecognised randomisation in the re-activation of quiescent BGCs. Access to these congeners has allowed us to characterise the effects of each modification on the bioactivity and clearly underline the importance of post-PKS tailoring reactions, particularly 18,19-epoxidation and *O*-acetylation. These congeners are now available for more in-depth mechanistic studies. Importantly, despite exhibiting higher MIC/IC_50_ values than **1**, these new congeners are equally selective activity against mammalian tumour cell lines and therefore may provide useful scaffolds for the generation of semi-synthetic therapeutics. In particular, compounds **3–6** offer scaffolds for launching a medicinal chemistry semi-synthesis approach to re-fashion an exciting natural product towards a new drug.

Understandably, many natural product discovery efforts focus on heterologous expression of BGCs. However engineering of producing strains remains a viable strategy for the discovery of new natural products. Due to their ubiquity and reliability, LALs have become an important tool to activate latent metabolic pathways in *Streptomyces* as shown by recent studies.^41,42^ In addition to activation of biosynthetic pathways, the overexpression of pathway specific regulators represents a strategy for the generation of analogues of known natural products, which provide important resources for understanding the structural–functional relationships of compound classes, the generation of variants with novel functions and elucidation of biosynthetic pathways.

## Materials and methods

For details regarding experimental procedures see the ESI.†

## Conflicts of interest

The authors declare no competing interest.

## Supplementary Material

SC-011-D0SC01928C-s001
