# Supplementary material for: Production of novel pladienolide analogues through native expression of a pathway-specific activator
Source: Chem Sci. 2020 Jul 17;11(31):8249–55. doi: 10.1039/d0sc01928c (PMC8163091; doi:10.1039/d0sc01928c)
Supplement: SC-011-D0SC01928C-s001 [file SC-011-D0SC01928C-s001.pdf]

Electronic Supplementary Material (ESI) for Chemical Science.
This journal is © The Royal Society of Chemistry 2020

Production of novel pladienolide analogues through native expression of a pathway specific activator

Thomas J. Booth[a], John A. Kalaitzis[b], Daniel Vuong[c], Andrew Crombie[c], Ernest Lacey[c*], Andrew M. Piggott[b]* and Barrie Wilkinson[a]*

[a] Department of Molecular Microbiology, John Innes Centre, Norwich Research Park, Norwich NR4 7UH, UK

[b] Department of Molecular Sciences, Macquarie University, NSW 2109, Australia

[c] Microbial Screening Technologies, Smithfield, NSW 2164, Australia

* To whom correspondence should be addressed.

Email: barrie.wilkinson@jic.ac.uk; andrew.piggot@mq.edu.au; elacey@microbialscreening.com

Contents

1. Supplementary Methods 4

1.1 Strains and Culture Conditions 4

1.2 Extraction and Sequencing of Genomic DNA 4

1.3 Molecular Cloning and Transformation 5

1.4 Screening for Pladienolide Production 6

1.5. Large Scale Culture and Isolation 7

1.6. Structural Elucidation 8

1.7. Assays for Biological Activity 9

2. Supplementary Figures 11

Figure S1: MLST Phylogeny of the Streptomyces platensis strains 11

Figure S2: Plasmid maps for a.) pGP9 and b.) pTJB1 12

Figure S3: HPLC trace of *Streptomyces platensis* AS6242 ethyl acetate extracts including pladienolide common chromophore. 13

Figure S4: Isolation scheme of the pladienolides from AS6200 pGP9+*pldR*: A.) **1** – **5** and B.) **6** 14

Figure S5. ^{1}H NMR spectrum (600 MHz, DMSO-d_6) of pladienolide B (**1**) 15

Figure S6. ^{13}C NMR spectrum (150 MHz, DMSO-d_6) of pladienolide B (**1**) 16

Figure S7. ^{1}H NMR spectrum (600 MHz, DMSO-d_6) of 6-deoxypladienolide (**2**) 17

Figure S8. ^{13}C NMR spectrum (150 MHz, DMSO-d_6) of 6-deoxypladienolide (**2**) 18

Figure S9. ^{1}H NMR spectrum (600 MHz, DMSO-d_6) of $\Delta^{18,19}$-pladienolide B (**3**) 19

Figure S10. ^{13}C NMR spectrum (150 MHz, DMSO-d_6) of $\Delta^{18,19}$-pladienolide B (**3**) 20

Figure S11. ^{1}H NMR spectrum (600 MHz, DMSO-d_6) of $\Delta^{18,19}$-6-deoxypladienolide B (**4**) 21

Figure S12. ^{13}C NMR spectrum (150 MHz, DMSO-d_6) of $\Delta^{18,19}$-6-deoxypladienolide B (**4**) 22

Figure S13. ^{1}H NMR spectrum (600 MHz, DMSO-d_6) of $\Delta^{18,19}$-6-deoxy-7-desacetylpladienolide B (**5**) 23

Figure S14. ^{13}C NMR spectrum (150 MHz, DMSO-d_6) of $\Delta^{18,19}$-6-deoxy-7-desacetylpladienolide B (**5**) 24

Figure S15. ^{1}H NMR spectrum (600 MHz, DMSO-d_6) of isopladienolide B (**6**) 25

Figure S16. ^{13}C NMR spectrum (150 MHz, DMSO-d_6) of isopladienolide B (**6**) 26

Figure S17: Inhibition of NS-1 by pladienolide B (**1**) and its analogues 27

Figure S18: Inhibition of NFF by pladienolide B (**1**) and its analogues 28

Figure S19: Inhibition of DU145 by pladienolide B (**1**) and its analogues...................................... 29

3. Supplementary Tables.. 29

Table S1: Plasmids used in this study 30

Table S2: Strains used in this study.. 31

Table S3: Genome Assembly Results 32

Table S4: Comparison of AS6200 and MA5455 contigs. Percentage pairwise identity/ percentage identical sites. 33

Table S5: Comparison of *pld* BGC between AS6200 and Mer-1107 34

Table S6: MIC of pladienolide analogues 35

Table S7: ^{1}H (600 MHz) and ^{13}C (150 MHz) NMR data for pladienolide B (**1**) in DMSO-d_6 36

Table S8: ^{1}H (600 MHz) and ^{13}C (150 MHz) NMR data for 6-deoxypladienolide (**2**) in DMSO-d_6 37

Table S9: ^{1}H (600 MHz) and ^{13}C (150 MHz) NMR data for □^{18,19}-pladienolide B (**3**) in DMSO-$d_6$38

Table S10: ^{1}H (600 MHz) and ^{13}C (150 MHz) NMR data for □^{18,19}-6-deoxypladienolide B (**4**) in DMSO-d_6.. 39

Table S11: ^{1}H (600 MHz) and ^{13}C (150 MHz) NMR data for □^{18,19}-6-deoxy-7-desacetylpladienolide B (**5**) in DMSO-d_6 40

Table S12: ^{1}H (600 MHz) and ^{13}C (150 MHz) NMR data for isopladienolide B (**6**) in DMSO-d_6... 41

4. References... 42

1. Supplementary Methods

1.1 Strains and Culture Conditions

A list of strains used in this study can be found in Table S1. *Streptomyces* spp. were maintained at 30 °C on SF+M (soy flour 20.0 g L^{-1}, mannitol 20.0 g L^{-1}) containing 20 % agar. *E. coli* were maintained at 37 °C on LB (NaCl 10.0 g L^{-1}, tryptone 10.0 g L^{-1}, yeast extract 5.0 g L^{-1}) containing 15% agar. Glycerol stocks were generated from a 1:1 mixture of liquid culture and 40% glycerol and stored at –80 °C. *Streptomyces* spore stocks were generated using a modified version of the protocol published by Kieser et al.[1]. Spores were harvested in sterile water (5 mL) and filtered through sterile cotton wool. Filtrate was centrifuged at 18,000 × *g*, the supernatant was discarded and spores were concentrated in 20% glycerol (100 µL).

Bacillus subtilis and *Staphylococcus aureus* were maintained at 28 °C on nutrient broth (5.0 g L^{-1} bacteriological peptone (Bacto), 5.0 g L^{-1} NaCl (Chem-supply), 2.0 gL^{-1} yeast extract (Bacto), 1.0 g L^{-1} beef extract (Bacto)). *Candida albicans* and *Saccharomyces cerevisiae* were maintained at 28 °C on malt extract broth (1.0 g L^{-1} bacteriological peptone (Bacto), 20.0 g L^{-1} malt extract (Bacto), 20 g L^{-1} glucose (Country Brewers)). Glycerol stocks were generated from 1:1 mixture of liquid culture and 40% glycerol and stored at -80 °C.

NS-1 murine myeloma cells (ATCC TIB-18), DU145 prostate cancer cells (ATCC HTB-81), and NFF (ATCC PCS-201) human neonatal foreskin fibroblast cells were maintained in Dulbecco's Modified Eagle Medium (DMEM) (Life Technologies Cat No. 10566016 + 10% v/v fetal bovine serum (FBS) (Life Technologies Cat. No. 16000044 + 1% v/v penicillin/streptomycin (10,000U mL^{-1}/10,000µg $mL^{-1)}$, Life Technologies Cat. No. 15140122) in 37 °C (5% CO_2) incubator. Cell lines were preserved in DMEM + FBS + Pen/Strep complete medium (90% v/v) + DMSO (10% v/v) and stored at -80 °C.

1.2 Extraction and Sequencing of Genomic DNA

High molecular weight genomic DNA was extracted according to the salting out procedure. Wet mycelium (0.5 mL) from 30-hour-old SV2 culture was washed with 10% sucrose (10 mL) before

resuspension in SET buffer (5 mL; 75 mM NaCl, 25 mM EDTA, 20 mM Tris HCl pH 8.0) to which lysozyme (200 µL; 50 mg mL^{-1}) and ribonuclease A (15 µL; 10 mg mL^{-1}) were added. The cells were incubated overnight at 37 °C; fresh lysozyme (300 µL) was added after ca. 17 h followed by an additional 2 h incubation. The subsequent steps were performed according to Kieser et al.[1].

Genomic DNA of *Streptomyces platensis* AS6200 and *Streptomyces platensis* MA5455 were sequenced using Pacific Biosciences (PacBio) RSII SMRT technology (commissioned to The Genome Analysis Centre (TGAC) Norwich, UK)[2] and assembled *via* the HGAP2.0 pipeline. Detection of specialised metabolic BGCs and annotation of gDNA was performed in antiSMASH v4.0[3] including ClusterBlast and whole-genome PFAM analysis[4]. Gene homologues were identified from translated protein sequences using BLASTp[5]. The BGC sequence was deposited into the GenBank database under the accession: MN974405. Whole genome alignments were performed using LASTZ[6]. Genomes were submitted to autoMLST[7] for taxonomic classification. The resulting alignment (95,515 bp) was used to generate a phylogenetic tree using FastTree 2[8].

1.3 Molecular Cloning and Transformation

The positive regulator *pldR* was amplified from *Streptomyces platensis* AS6200 genomic DNA *via* polymerase chain reaction (PCR) using the primers pldR_F (ttaattaaggaggacacatatgcatctcttcgggcgggac) and pldR_R (ccaagctcagctaattaagcttcatgccgtcggggcag) and Q5 high-fidelity DNA polymerase in 25 – 50 µL aliquots according to the manufacturer's protocol supplemented with 3% DMSO. For screening purposes, PCR was performed using GoTaq Green Master Mix (Promega) in 10 µL reactions. Thermal cycling was carried out according to manufacturer's protocol (initial denaturation at 98 °C for 30 s followed by 35 cycles of denaturation at 98 °C for 10 s, annealing at 60 °C for 30 s and extension at 72 °C for 1 min 30 s, followed by a final extension at 72 °C for 2 min). The plasmid pGP9 was digested with *Nde*I and *Hin*dIII (New England Biolabs) according to manufacturer's protocols. PCR and digestion products were purified using Wizard SV gel and PCR clean up system (Promega) according to manufacturer's protocols. Molecular cloning was performed *via* Gibson Assembly[9] in 10 µL reactions using Gibson Assembly MasterMix (New England Biolabs) according to the manufacturer's

protocols. Plasmids were transformed into electrocompetent *E. coli* DH5α using a BioRad GenePulser (2.5 kV, 200 Ω, 25 µF). Resulting plasmids were confirmed by Sanger sequencing (Mix2Seq, Eurofins Genomics) with the primers pGP9_F (gagcggcggtcgaagggagatg) and pGP9_R (cgagcgttctgaacaaatccag).

Plasmids were extracted from *E. coli* DH5α using Wizard Plus SV miniprep system (Promega) and transformed into electrocompetent *E. coli* ET12567 pUZ8002 as above. The resulting strains were used for conjugation with *S. platensis* AS6200 spores according to Kieser et al.[1].

1.4 Screening for Pladienolide Production

The exconjugants *S. platensis* AS6200 pGP9+*pldR* and *S. platensis* AS6200 pGP9 were screened on the following media: SV2 (glucose 15.0 g L^{-1}, glycerol 15.0 g L^{-1}, soy peptone 15.0 g L^{-1}, NaCl 3.0 g L^{-1}, $CaCO_3$ 1.0 g L^{-1}), MYM (maltose 4.0 g L^{-1}, yeast extract 4.0 g L^{-1}, malt extract 10.0 g L^{-1}), SF+M, PYE (peptone 2.0 g L^{-1}, yeast extract 1.0 g L^{-1}, $CaCl_2$ 0.07 g L^{-1}) and SM6 (corn steep liquor 40 g L^{-1}, maltodextrin 20.0 g L^{-1}, NaCl 2.5 g L^{-1}, $MgSO_4$ 0.5 g L^{-1}), SM14 (glucose 10.0 g L^{-1}, Bacto Soytone 20.0 g L^{-1}, meat extract 5.0 g L^{-1}, NaCl 5.0 g L^{-1}, $ZnSO_4 \cdot 2H_2O$ 0.01 g L^{-1}) and SM18 (glucose 15.0 g L^{-1}, soluble starch 25.0 g L^{-1}, Pharmamedia 25.0 g L^{-1}, cane molasses 20.0 g L^{-1}, $CaCO_3$ 8.0 g L^{-1}) containing 200 µg/mL apramycin. Cultures (25 mL) were grown for 7 days at 30 °C. An aliquot (1 mL) of each culture was mixed 1:1 with ethyl acetate at room temperature for 10 min. Following incubation, the organic phase was removed and dried *via* evaporation and the residue was dissolved in methanol (100 µL). Samples were analysed by LCMS. LCMS was performed on a Shimadzu LC–MS platform (equipped with a NexeraX2 liquid chromatograph (LC30AD), a Prominence photodiode array detector (SPD-M20A) and an LCMS-IT-TOF mass spectrometer) with chromatography over a Kinetex C_{18} 100 Å column (100 × 2.1 mm, 2.6 µm; Phenomenex) using a water-methanol gradient (20-100% methanol over 12 min with 1 min hold at 100%). Pladienolide B was identified from the calculated mass.

1.5. Large Scale Culture and Isolation

Cultures were grown in SM18 liquid media with 1% C_{18} resin (Grace Discovery) in 250 mL Erlenmeyer flasks each containing 50 mL media. A spore suspension of a 7-day-old Petri plate of *Streptomyces platensis* AS6242 pGP9+*pldR* was used to inoculate 77 × 250 mL flasks. Ferment A (77 × 50 mL) was incubated at 28 °C for 14 days and Ferment B (77 x 50 ml) was incubated at 24 °C for 14 days. Each culture was pooled and centrifuged, following which the pelleted mycelia and resin were extracted with methanol (2 × 2 L) and evaporated *in vacuo* to produce an aqueous slurry (500 mL). The slurry was partitioned against ethyl acetate (2 × 1 L) and the ethyl acetate was reduced *in vacuo* to give the crude extract (Culture A 2.5 g, Culture B 1.4 g).

Analytical HPLC was performed on a gradient Agilent 1260 Infinity quaternary HPLC system. The column was an Agilent Zorbax SB-C18 (2.1 × 50 mm, 1.8 µm) eluted with a 0.6 mL min^{-1} gradient of 10-100% MeCN/H_2O (0.01% TFA) over 11 min. Preparative HPLC was performed on a gradient Shimadzu HPLC system comprising two LC-8A preparative liquid pumps with a static mixer, SPD-M10AVP diode array detector and SCL-10AVP system controller with a standard Rheodyne injection port. The column was a Zorbax SB-C18 column (50 × 150 mm, 5 µm; Agilent) eluted isocratically at 60 mL/min. Further purification was undertaken using a semi-preparative Zorbax SB-C18 column (2 × 250 mm, 5 µm; Agilent) eluted isocratically at 20 mL min^{-1}.

The crude extract from Culture A was dissolved with methanol and fractionated by preparative HPLC (Zorbax C18, isocratic 45% MeCN/H_2O, 60 mL min^{-1}) to give two enriched fractions. Fraction 1 (87.2 mg) was purified by isocratic preparative HPLC (Zorbax C18, isocratic 45% MeCN/H_2O, 20 mL min^{-1}) to yield **1** (t_R 11.32 min; 31.9 mg). Fraction 2 (486.8 mg) was separated using a size exclusion chromatography column (25 × 600 mm) of Sephadex LH-20 resin (Davisil, Grace Discovery) in HPLC grade methanol. Fractions (4 mL) were collected at a flow rate of 4 mL min^{-1}. Fractions were analysed by C18 HPLC and three fractions (12 mL) were pooled and evaporated to yield a pladienolide enriched fraction. The enriched fraction was purified by isocratic preparative HPLC (Zorbax C18, isocratic stepwise 70% - 80% MeCN/H_2O, 20 mL min^{-1}) to yield **3** (t_R 9.73 min; 6.2 mg), **4** (t_R 10.91 min; 6.4

mg), **5** (t_R 11.97 min; 3.9 mg) and **2** (t_R 16.39 min; 3.0 mg). The crude extract from Culture B was dissolved with methanol and fractionated by preparative HPLC (Zorbax C18, isocratic 55% MeCN/H_2O, 60 mL min^{-1}) to give an enriched fraction, which was further purified by isocratic preparative HPLC (Zorbax C18, isocratic 45% MeCN/H_2O, 20 mL min^{-1}) to yield **6** (t_R 19.91 min; 2.1 mg).

1.6. Structural Elucidation

Careful analyses of 1D and 2D NMR data, recorded in DMSO-d_6 at 600 MHz (Tables S7 - S9), together with high resolution mass spectrometry data confirmed the structures of the previously reported metabolites pladienolide B (**1**), 6-deoxypladienolide B (**2**) and $\Delta^{18,19}$-pladienolide B (**3**)[10,11]

HRESI(+)MS analysis of $\Delta^{18,19}$-6-deoxypladienolide B (**4**) revealed a protonated molecule (*m/z* 505.3516 $[M+H]^+$, calculated 505.3524, Δ mmu –0.8) indicative of a molecular formula $C_{30}H_{48}O_6$, which is one oxygen atom less than both **2** and **3**. The ^1H and ^{13}C NMR data for **4** (Table S10) were very similar to those for **3**, with the only significant differences being the absence of a signal associated with the tertiary alcohol 6-OH (δ_H 4.52, s), the presence of an additional aliphatic methine signal (δ_H 1.80, m) and less deshielding of C-6 (δ_C 34.5 in **4** vs. δ_C 71.9 in **3**). Detailed analysis of the 2D NMR data (Table S10) confirmed **4** to be the 6-deoxy analogue of **3**.

HRESI(+)MS analysis of $\Delta^{18,19}$-6-deoxy-7-desacetylpladienolide B (**5**) revealed a protonated molecule (*m/z* 463.3410 $[M+H]^+$, calculated 463.3418, Δ mmu –0.8) indicative of a molecular formula $C_{28}H_{46}O_5$, which is C_2H_2O less than **4**. The ^1H and ^{13}C NMR data for **5** (Table S11) were very similar to those for **4**, with the only significant differences being the absence of signals associated with the 7-acetyl group (δ_H 1.97, s; δ_C 21.0 and 169.6), the presence of an additional exchangeable proton (δ_H 4.55, d) and less deshielding of H-7 (δ_H 3.36 in **5** vs. δ_H 4.78 in **4**). Detailed analysis of the 2D NMR data (Table S11) confirmed **5** to be the 7-desacetyl analogue of **4**.

HRESI(+)MS analysis of isopladienolide B (**6**) revealed an adduct ion (*m/z* 559.3234 $[M+Na]^+$, calculated 559.3241, Δ mmu –0.7) consistent with a molecular formula $C_{30}H_{48}O_8$, which is isomeric

with **1**. The ^{1}H and ^{13}C NMR data for **6** (Table S12) were very similar to those for **1**, with the main differences being located on the side chain and not the macrocyclic core. Most notably, the ^{1}H and ^{13}C resonances for H-18/C-18 and H-19/C-19 were significantly more deshielded in **6** (δ_H 3.57; δ_C 79.2 and δ_H 3.80; δ_C 77.5) than in **1** (δ_H 2.62; δ_C 56.0 and δ_H 2.55; δ_C 60.8), suggesting opening of the epoxide ring by an oxygen nucleophile. Diagnostic ^{1}H-^{13}C HMBC correlations from H-21 to C-18 and from to H-18 to C-21 were indicative of an intramolecular 5-*exo*-tet cyclisation from 21-OH to C-18 to form a tetrahydrofuran ring system. Detailed analysis of the 2D NMR data (Table S12) confirmed **6** to be the cyclised analogue of **1**.

1.7. Assays for Biological Activity

Purified metabolites (**1** – **6**) were dissolved in DMSO to provide stock solutions (1,000 µg/mL or 40 µg/mL). Bioassays were carried out in 96-well microtitre plates containing two-fold serial dilutions to yield ranges of 10 to 0.005 µg mL^{-1} or 0.4 to 0.0002 µg mL^{-1}, in 1% DMSO. Wells containing no test compound as a reference for no inhibition, and uninoculated wells were used as a reference for complete inhibition.

Antibacterial assays were carried out using *Bacillus subtilis* ATCC 6633 and *Staphylococcus aureus* ATCC 25923 as indicative species for Gram-positive activity. A bacterial suspension (50 mL in 250 mL flask) was prepared in nutrient broth media (5.0 g L^{-1} bacteriological peptone (Bacto), 5.0 g L^{-1} NaCl (Chem-supply), 2.0 g L^{-1} yeast extract (Bacto), 1.0 g L^{-1} beef extract (Bacto)) by cultivation for 24 h at 250 rpm, 28 °C. The suspension was diluted to an absorbance of 0.01 absorbance units per mL [OD400 nm] and aliquots (10 µL) were added to the wells of a 96-well microtitre plate containing the test compounds dispersed in nutrient broth with resazurin (12.5 µg mL^{-1}). The plates were incubated at 28 °C for 48 h, during which time the positive control wells change colour from a blue to light pink colour. MICs were determined visually.

Antifungal assays were carried out using *Candida albicans* ATCC 10231 and *Saccharomyces cerevisiae* ATCC 9763 as indicative species for antifungal activity. A yeast suspension (50 mL in 250 mL flask) was prepared in malt extract broth (1.0 g L^{-1} bacteriological peptone (Bacto), 20.0 g L^{-1} malt extract

(Bacto), 20 g L^{-1} glucose (Country Brewers)) by cultivation for 24 h at 250 rpm, 24 °C. The suspension was diluted to an absorbance of 0.005 and 0.03 absorbance units [OD_{400}] per mL for *C. albicans* and S. *cerevisiae*, respectively. Aliquots (20 µL and 30 µL) *of C. albicans* and S. *cerevisiae,* respectively were applied to the wells of a 96-well microtitre plate, which contained the test compounds dispersed in malt extract broth containing bromocresol green (50 µg mL^{-1}). The plates were incubated at 24 °C for 48 h during which time the positive control wells change colour from a blue to yellow colour. MIC end points were determined visually.

To assay antiprotozoal activity, *Tritrichomonas foetus* KV-1 was inoculated in 96-well microtitre plates (200 µL) at 4×10^4 cells/mL in TF medium (tryptone (Oxoid) 2 g L^{-1}, yeast extract 1 g L^{-1}, glucose 2.5 g L^{-1}, L-cysteine 1 g L^{-1}, K_2HPO_4 1 g L^{-1}, KH_2PO_4 1 g L^{-1}; ascorbic acid g L^{-1}; $FeSO_4 \cdot 7H_2O$ 0.1 g L^{-1}) supplemented with 1% v/v penicillin/streptomycin (10,000 U mL^{-1} / 10,000 µg mL^{-1}, Life Technologies Cat. No. 15140122), 10% v/v new born calf serum (NBCS), Life Technologies). Plates were incubated in anaerobic jars (Oxoid AG25) containing Anaerogen satchel (Oxoid AN25) at 37 °C (5% CO_2). *T. foetus* proliferation was measured at 72 h.

To assay antitumour activity, NS-1 ATCC TIB-18 mouse myeloma, DU145 prostate cancer cells (ATCC HTB-81), and NFF (ATCC PCS-201) human neonatal foreskin fibroblast cells were each inoculated in 96-well microtitre plates (190 µL) at 50,000 cells mL^{-1} in DMEM (Dulbecco's Modified Eagle Medium + 10% v/v fetal bovine serum (FBS) + 1% v/v penicillin/streptomycin (10,000U mL^{-1}/ 10,000 µg mL^{-1}, Life Technologies Cat. No. 15140122), together with resazurin (250 µg/mL; 10 µL) and incubated at 37 °C (5% CO_2). The plates were incubated for 96 h. Absorbance was measured using a Spectromax plate reader (Molecular Devices) at 605 nm for % inhibition, and IC_{50} values determined.

2. Supplementary Figures

Figure S1: MLST Phylogeny of the Streptomyces platensis strains

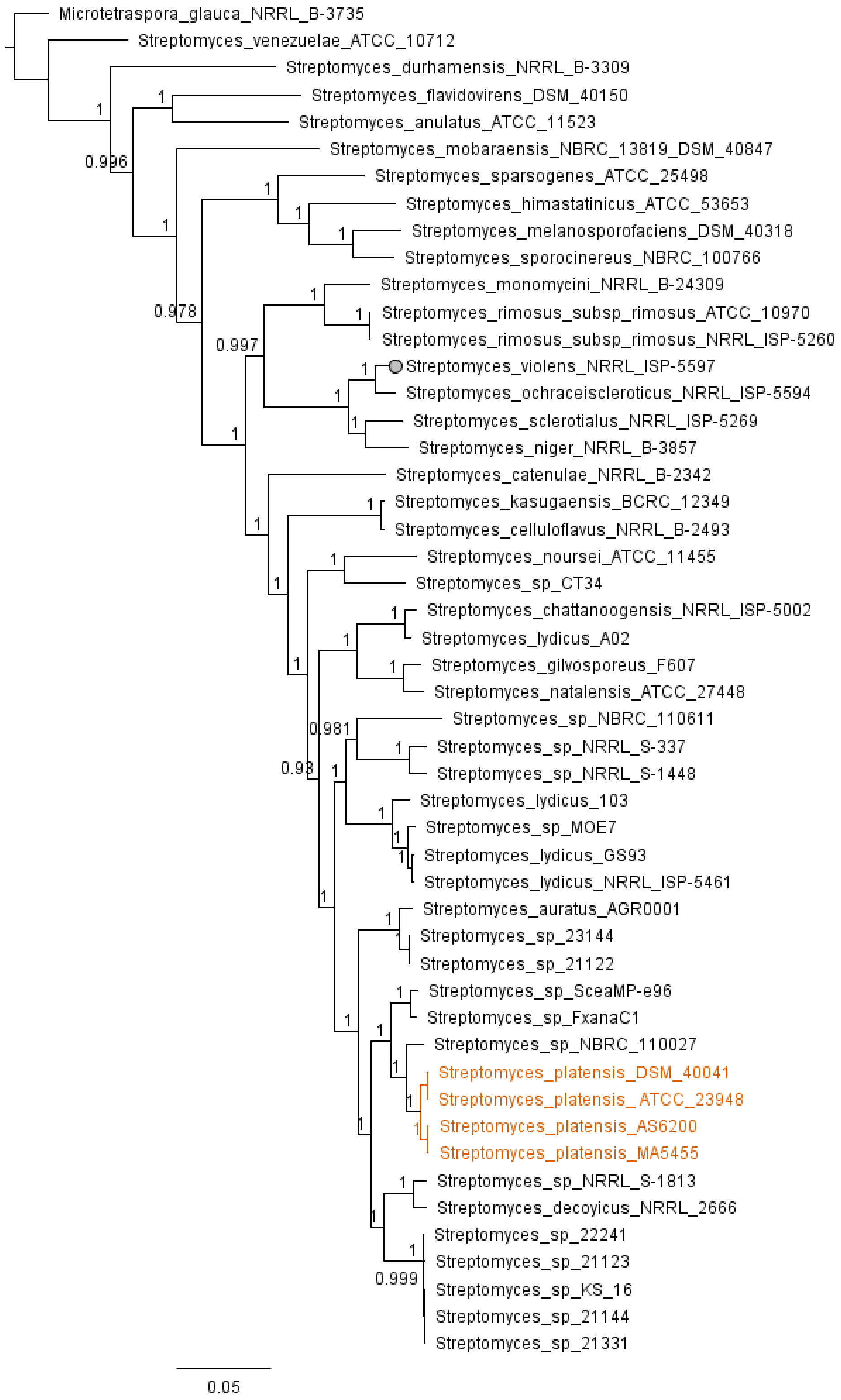

Figure S2: Plasmid maps for a.) pGP9 and b.) pTJB1

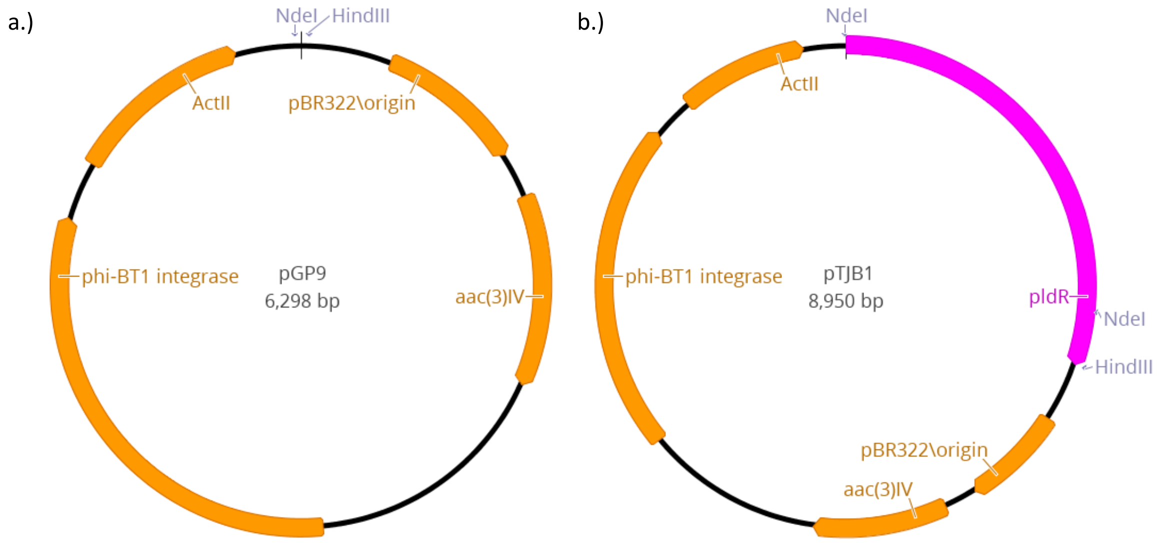

Figure S3: HPLC trace of *Streptomyces platensis* AS6242 ethyl acetate extracts measuring absorbance at 220 nm including pladienolide common chromophore.

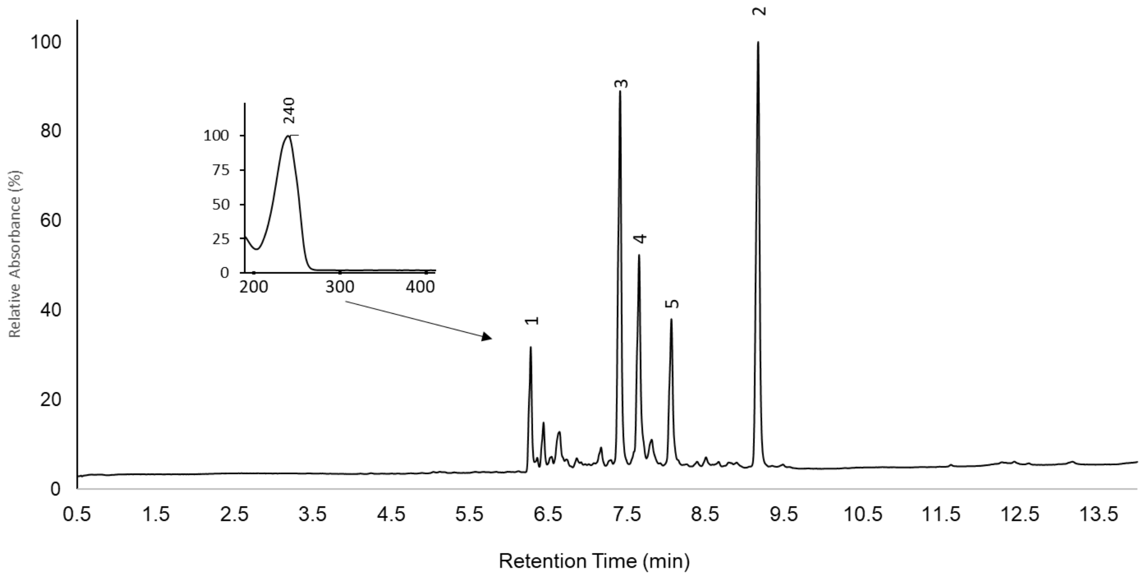

Figure S4: Isolation scheme of the pladienolides from AS6200 pGP9+*pldR*: A.) **1** – **5** and B.) **6**

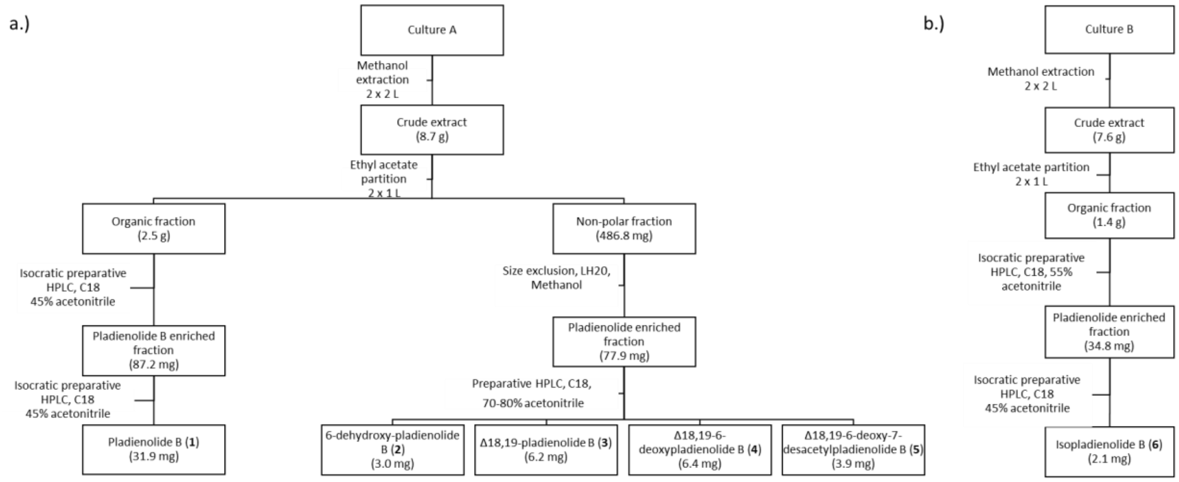

Figure S5. ^{1}H NMR spectrum (600 MHz, DMSO-d_6) of pladienolide B (**1**)

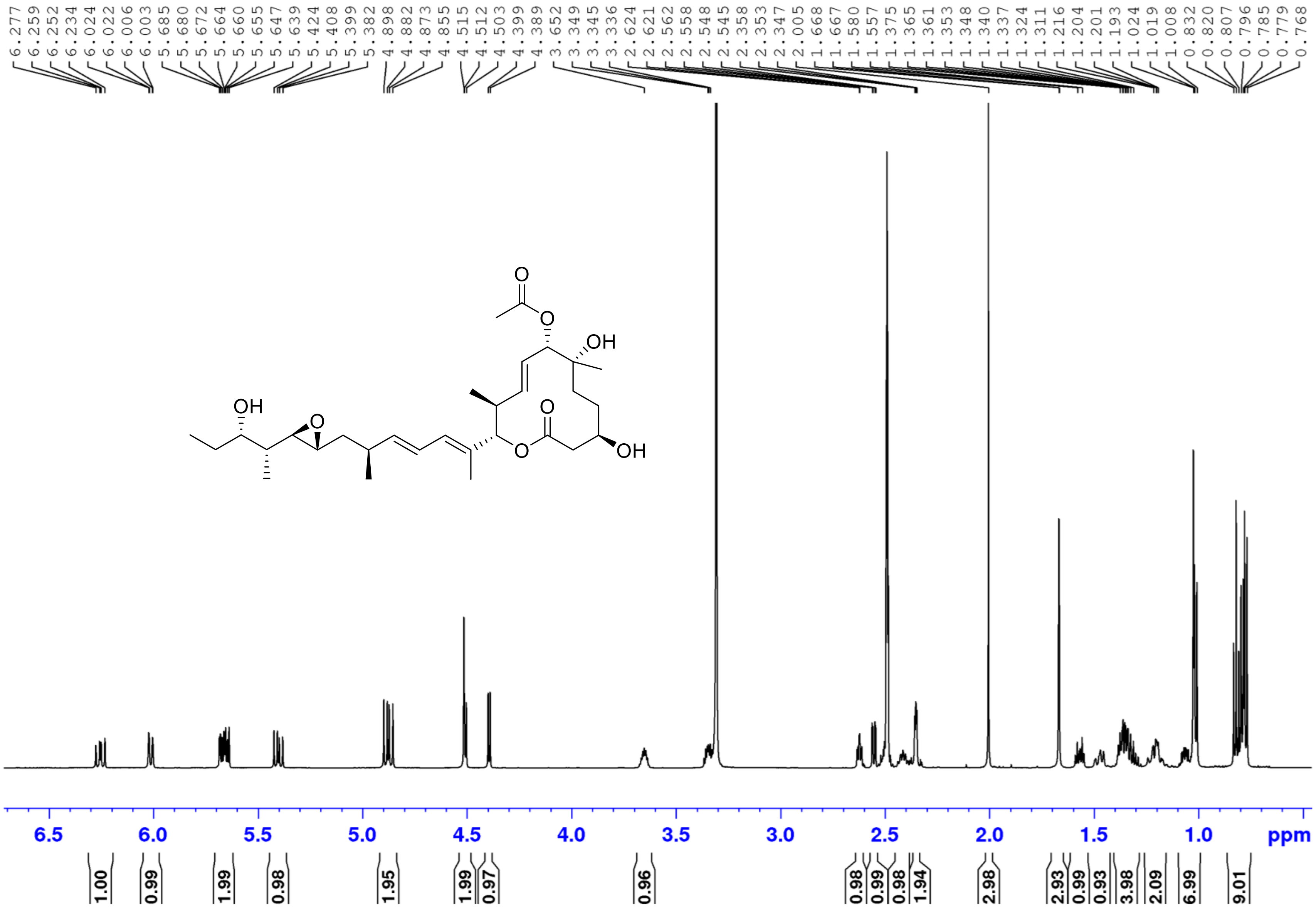

Figure S6. ^{13}C NMR spectrum (150 MHz, DMSO-d_6) of pladienolide B (**1**)

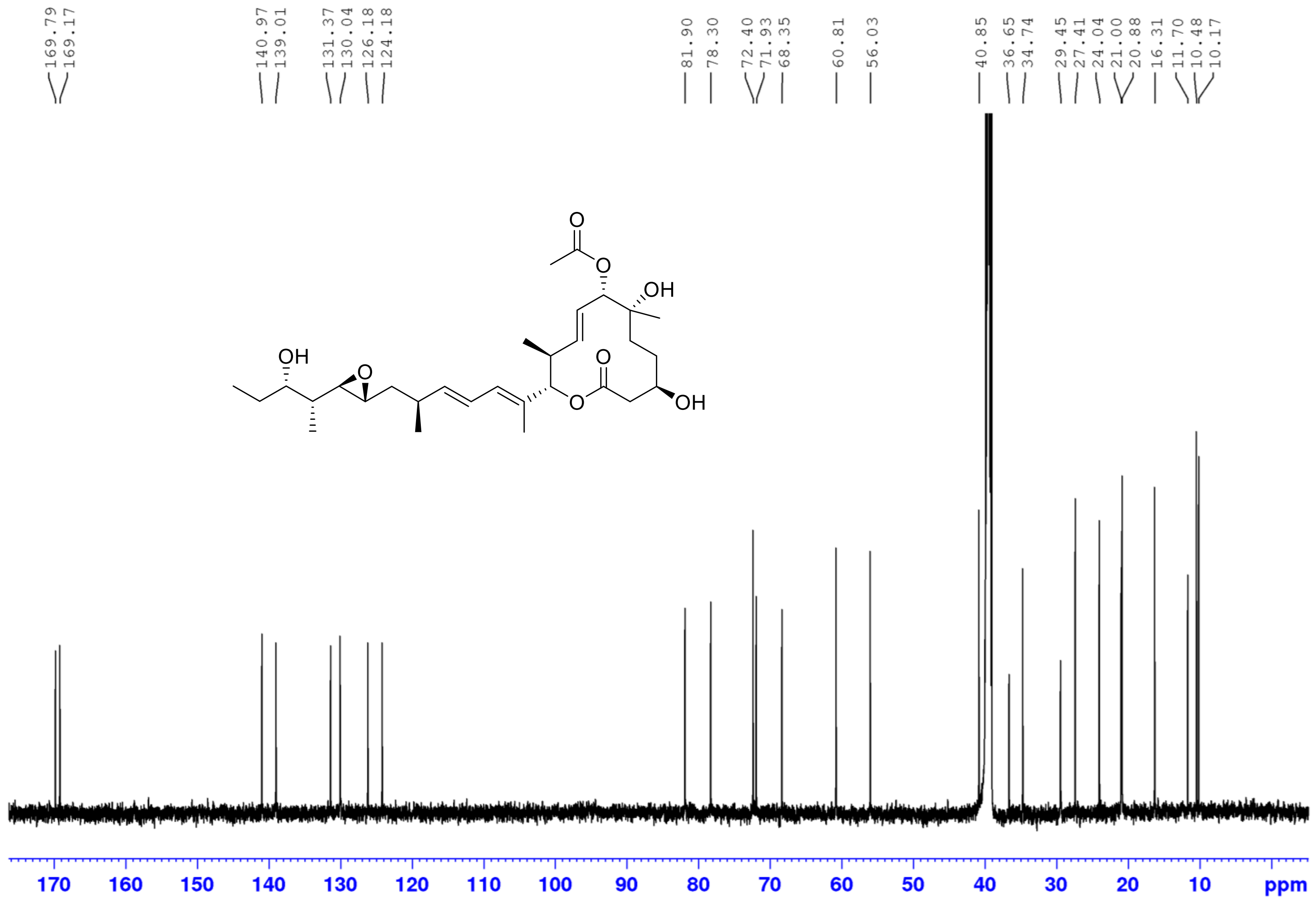

Figure S7. ^{1}H NMR spectrum (600 MHz, DMSO-d_6) of 6-deoxypladienolide (**2**)

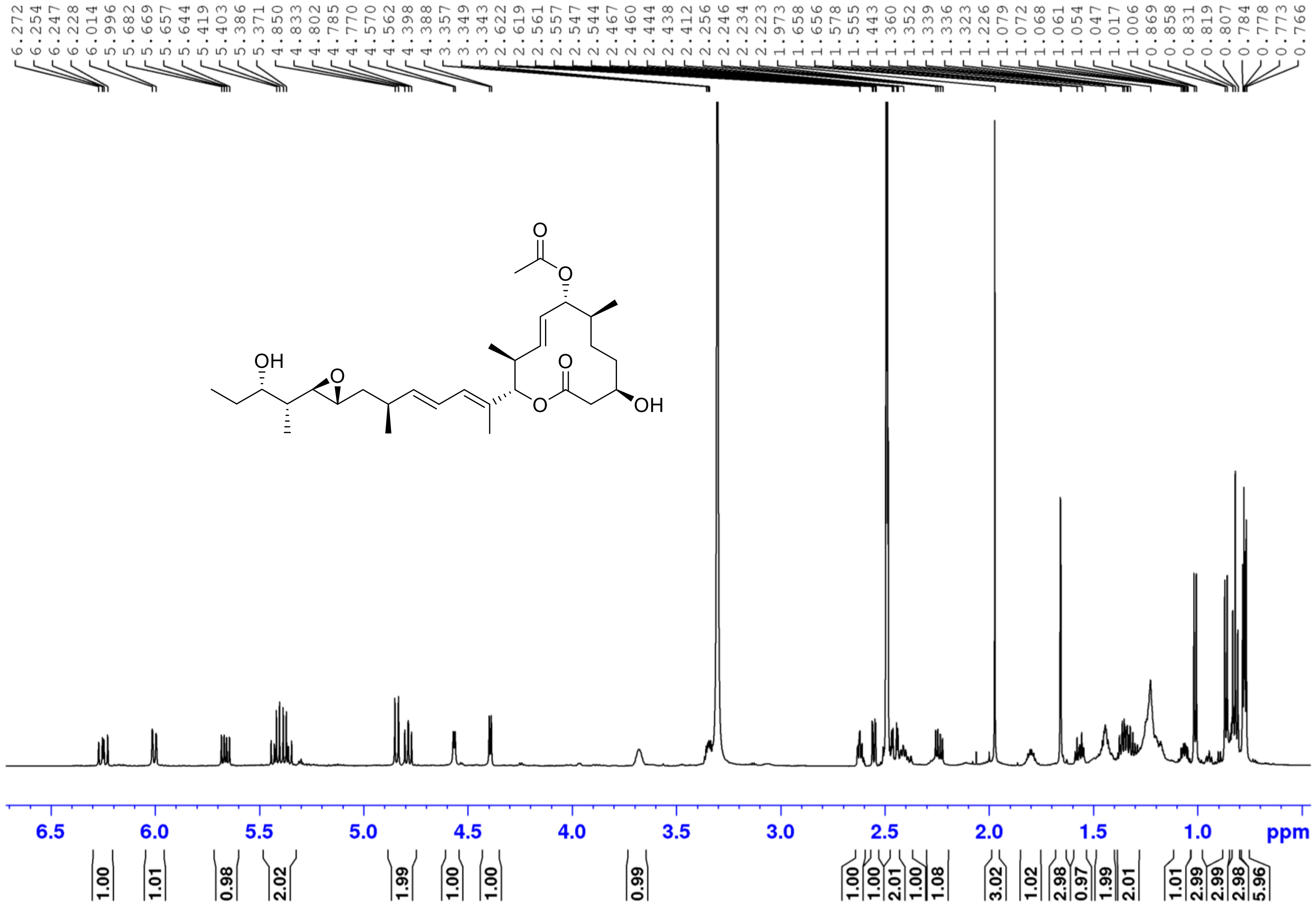

Figure S8. ^{13}C NMR spectrum (150 MHz, DMSO-d_6) of 6-deoxypladienolide (**2**)

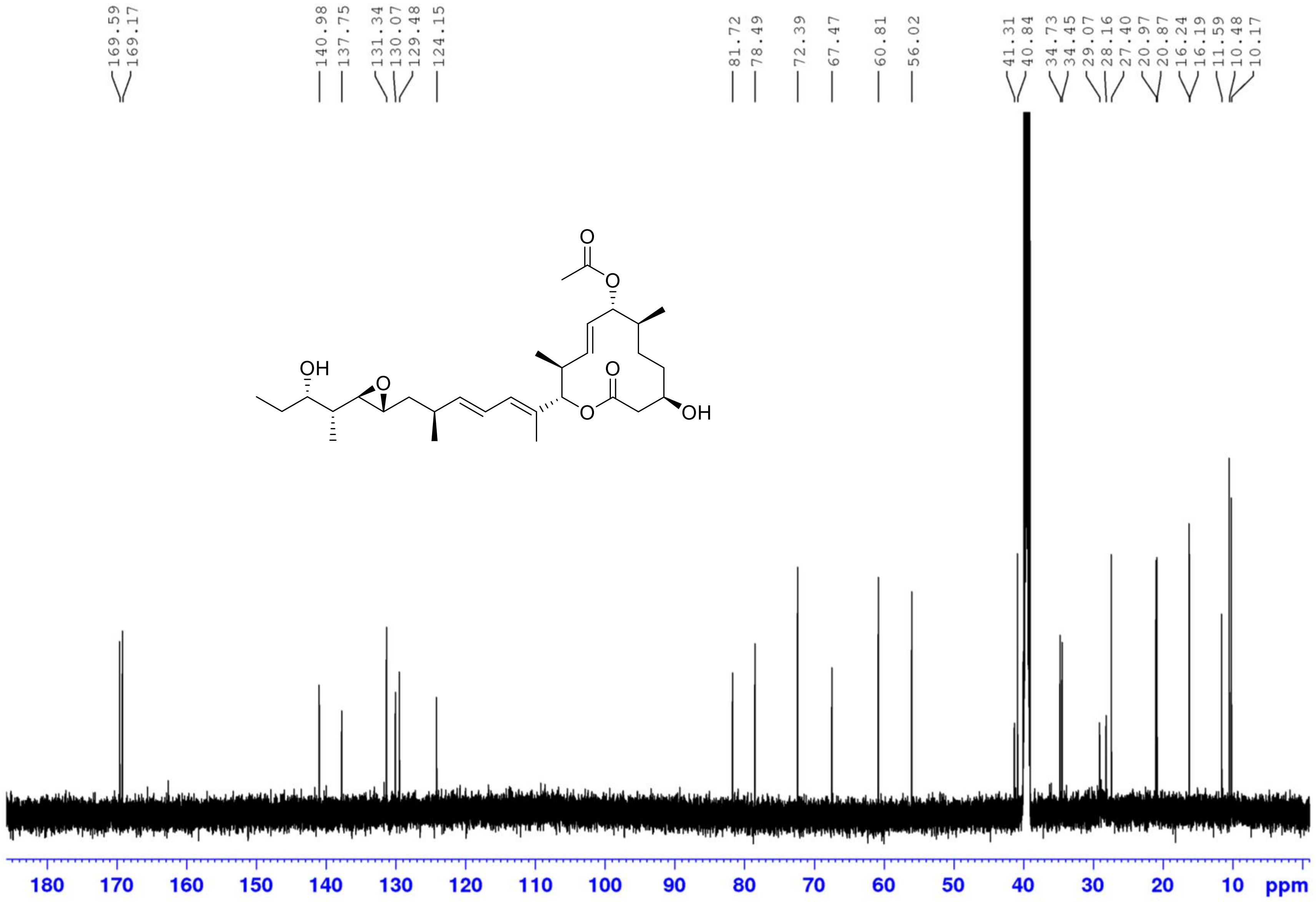

Figure S9. ^{1}H NMR spectrum (600 MHz, DMSO-d_6) of $\Delta^{18,19}$-pladienolide B (**3**)

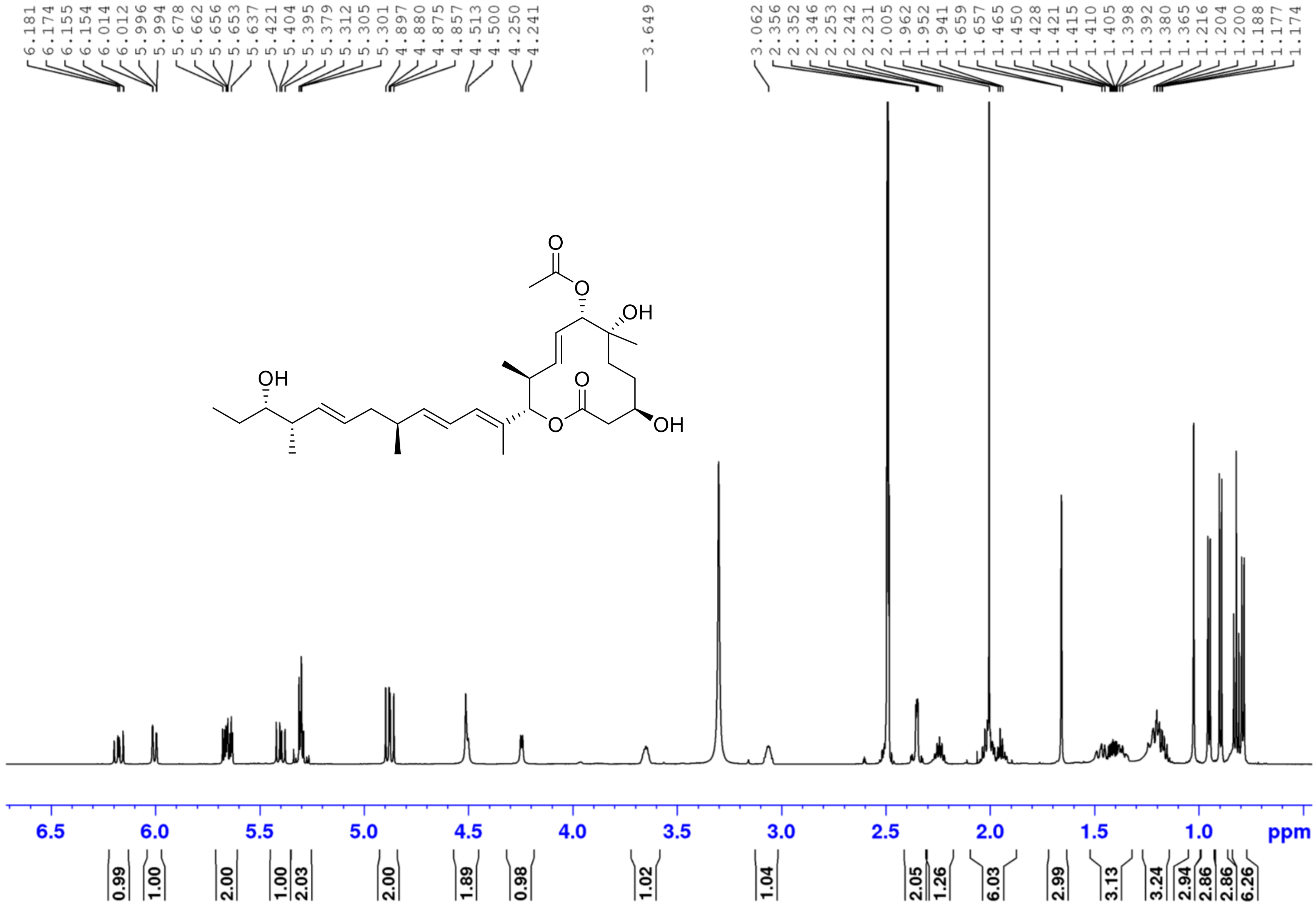

Figure S10. ^{13}C NMR spectrum (150 MHz, DMSO-d_6) of $\Delta^{18,19}$-pladienolide B (**3**)

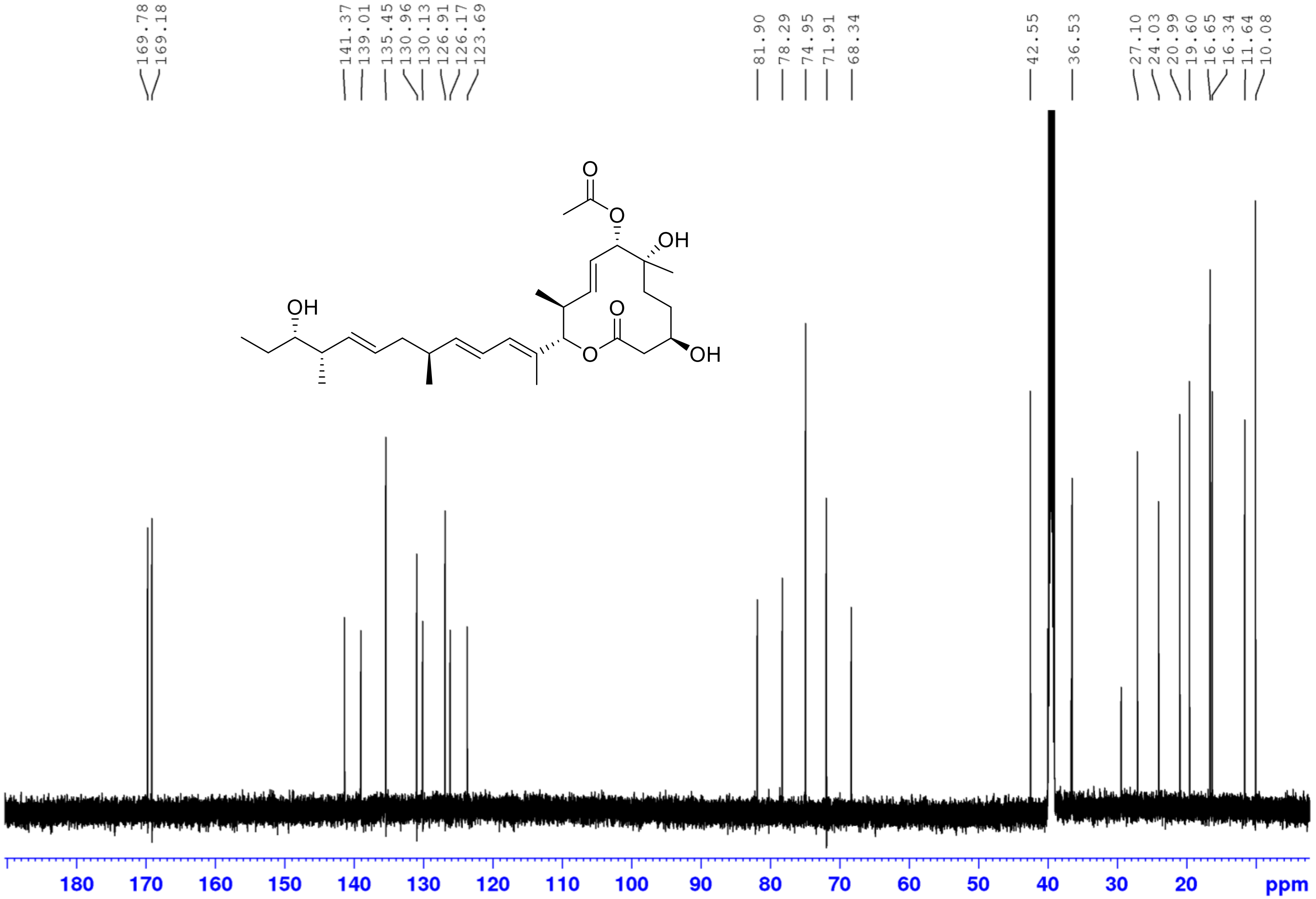

Figure S11. ^{1}H NMR spectrum (600 MHz, DMSO-d_6) of $\Delta^{18,19}$-6-deoxypladienolide B (**4**)

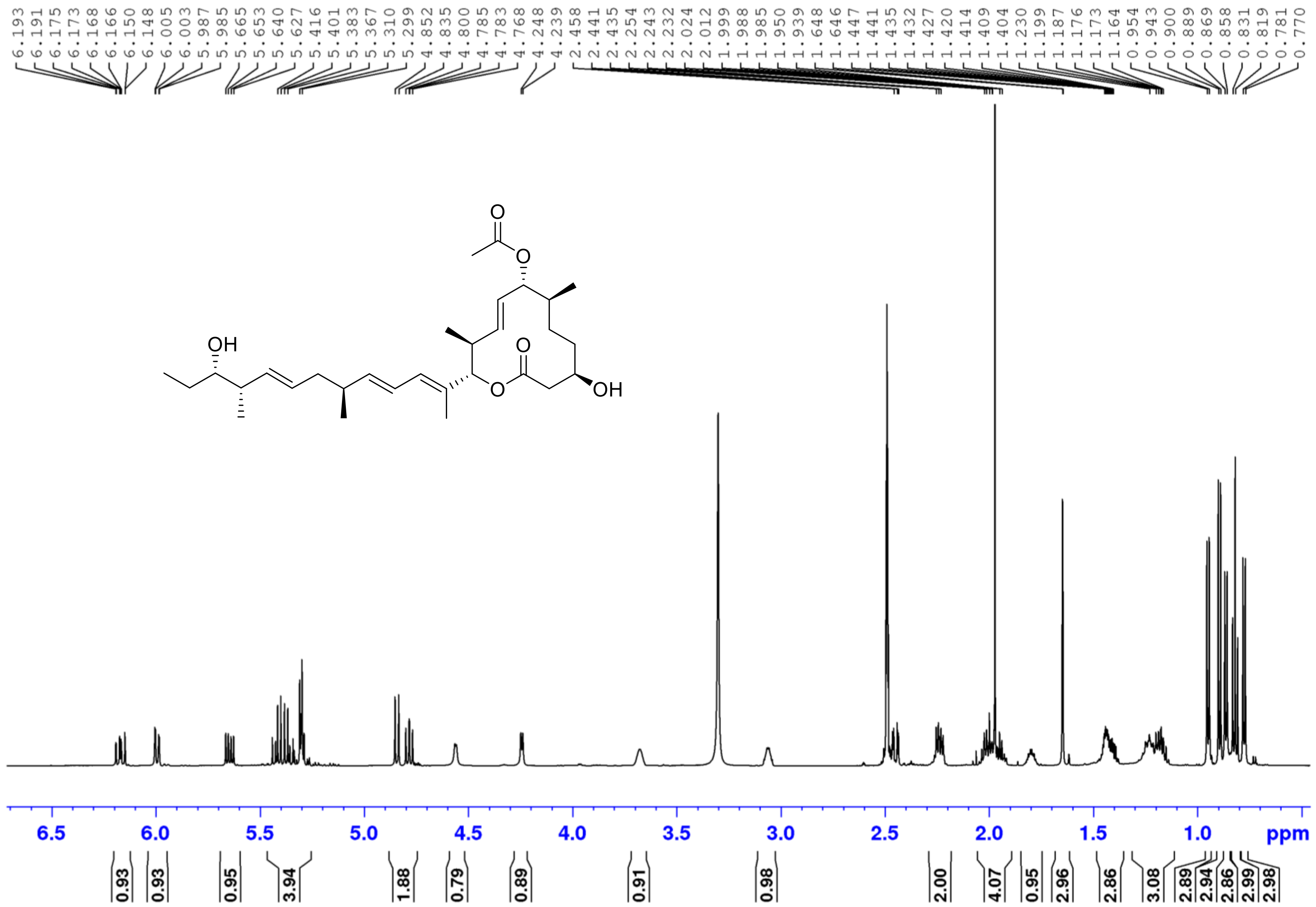

Figure S12. ^{13}C NMR spectrum (150 MHz, DMSO-d_6) of $\Delta^{18,19}$-6-deoxypladienolide B (**4**)

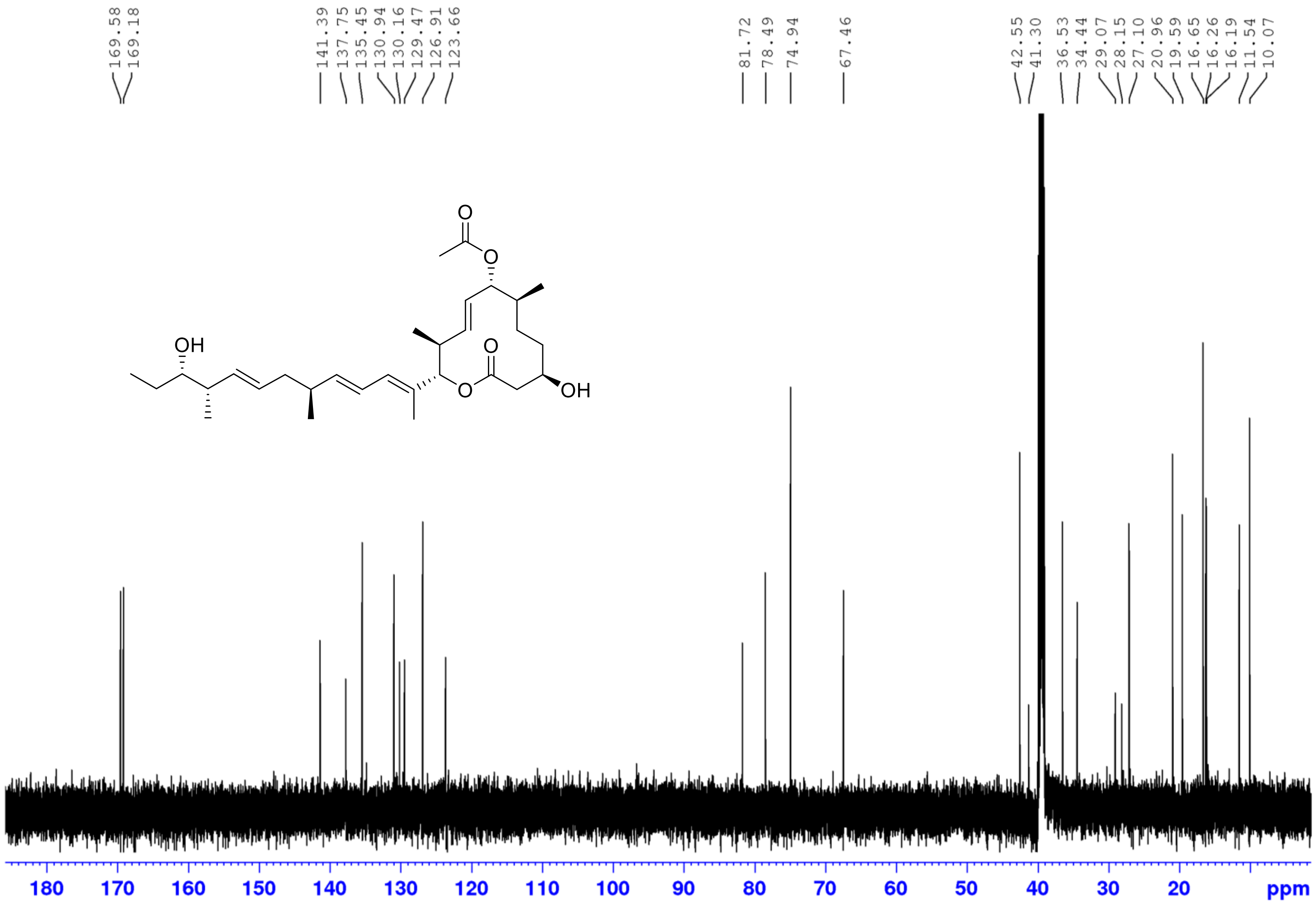

Figure S13. ^{1}H NMR spectrum (600 MHz, DMSO-d_6) of $\Delta^{18,19}$-6-deoxy-7-desacetylpladienolide B (**5**)

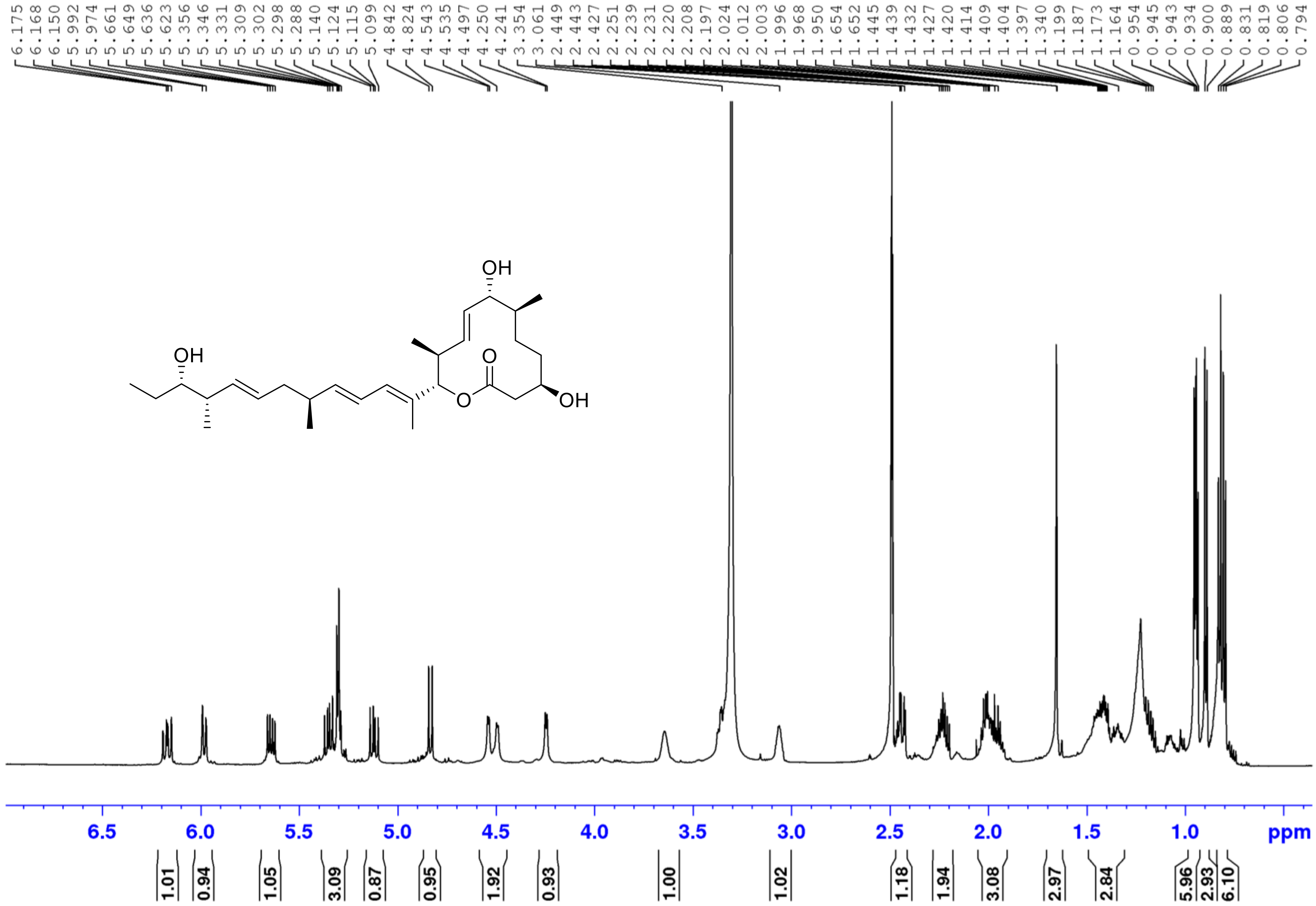

Figure S14. ^{13}C NMR spectrum (150 MHz, DMSO-d_6) of $\Delta^{18,19}$-6-deoxy-7-desacetylpladienolide B (**5**)

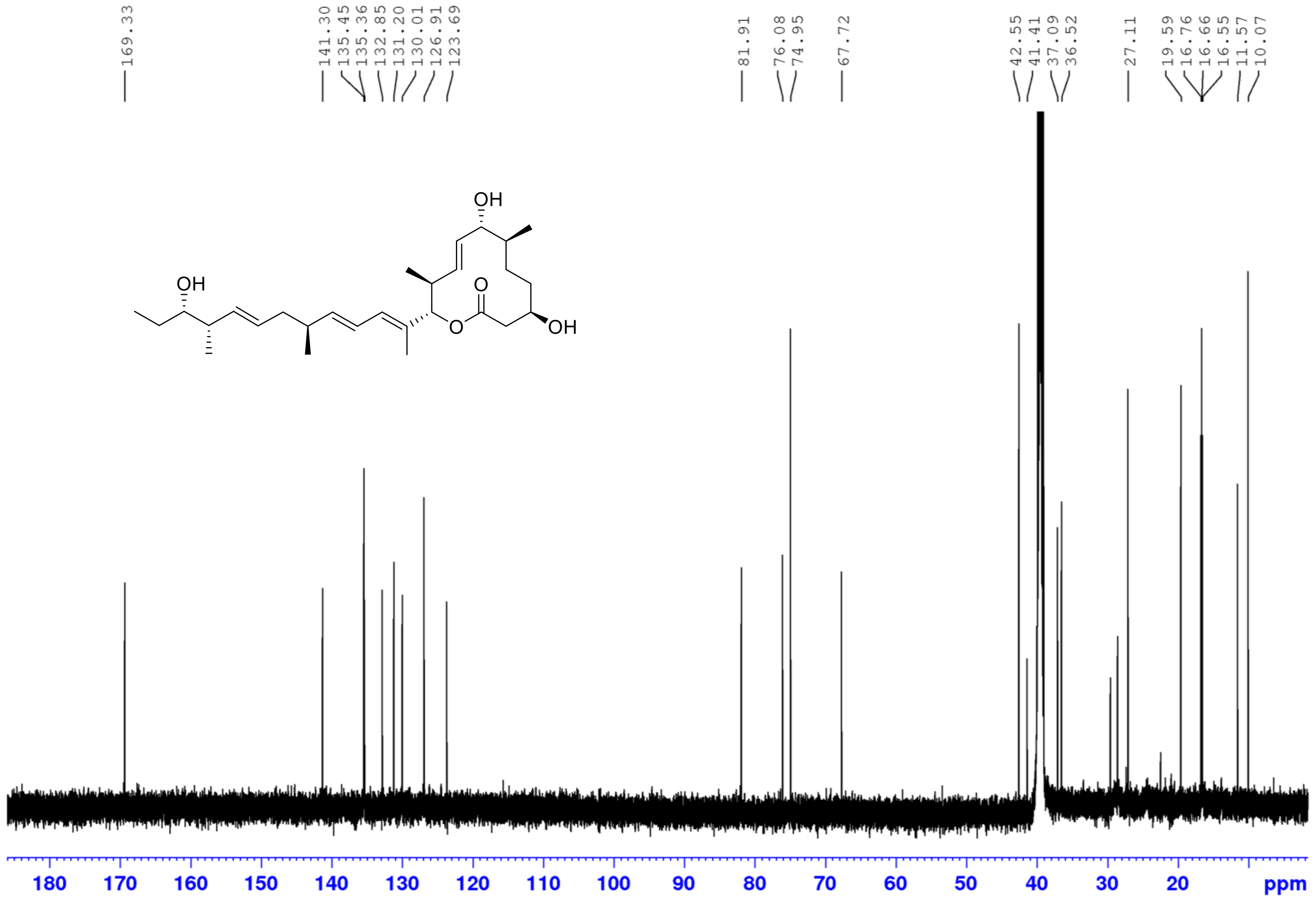

Figure S15. ^{1}H NMR spectrum (600 MHz, DMSO-d_6) of isopladienolide B (**6**)

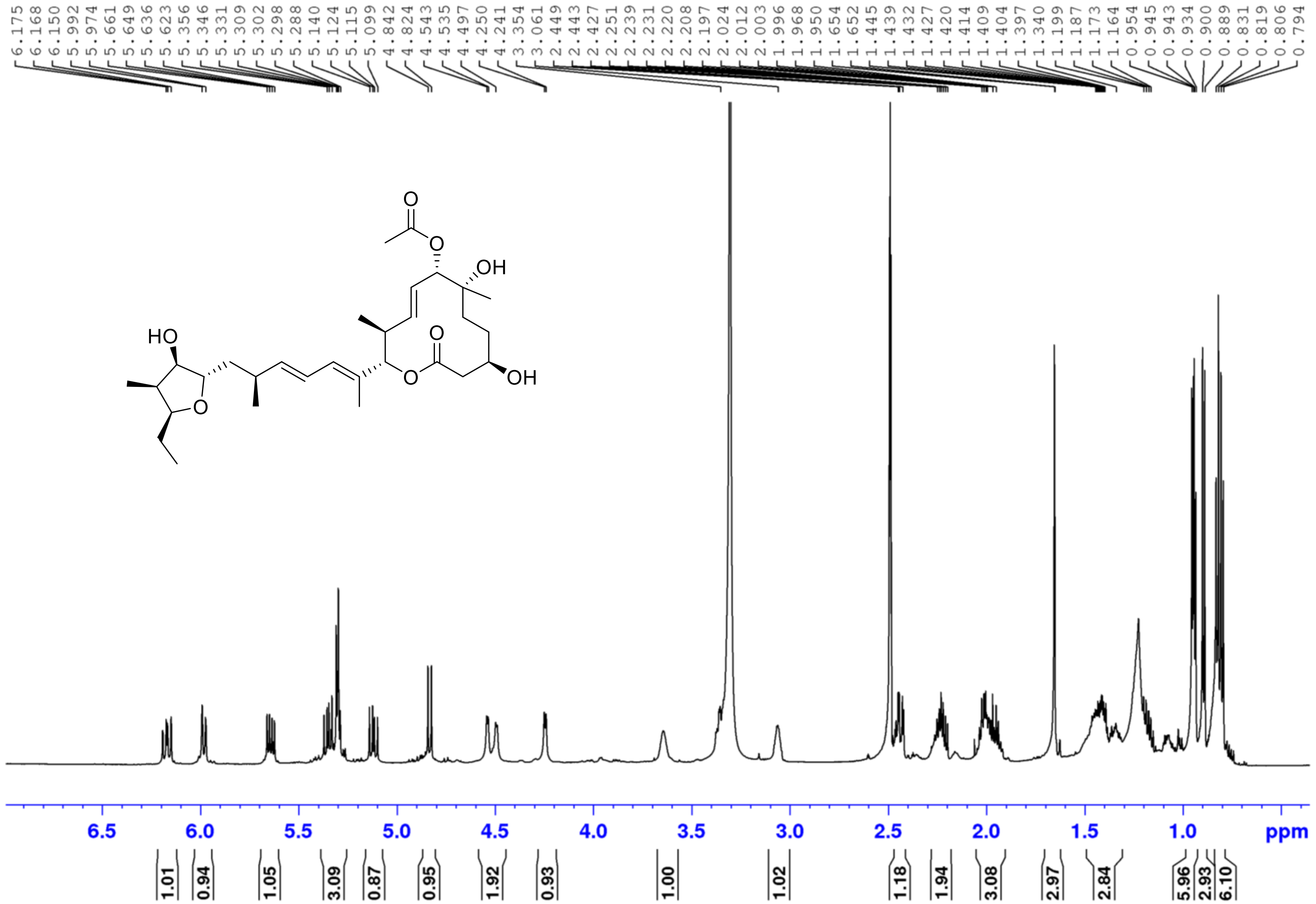

Figure S16. ^{13}C NMR spectrum (150 MHz, DMSO-d_6) of isopladienolide B (**6**)

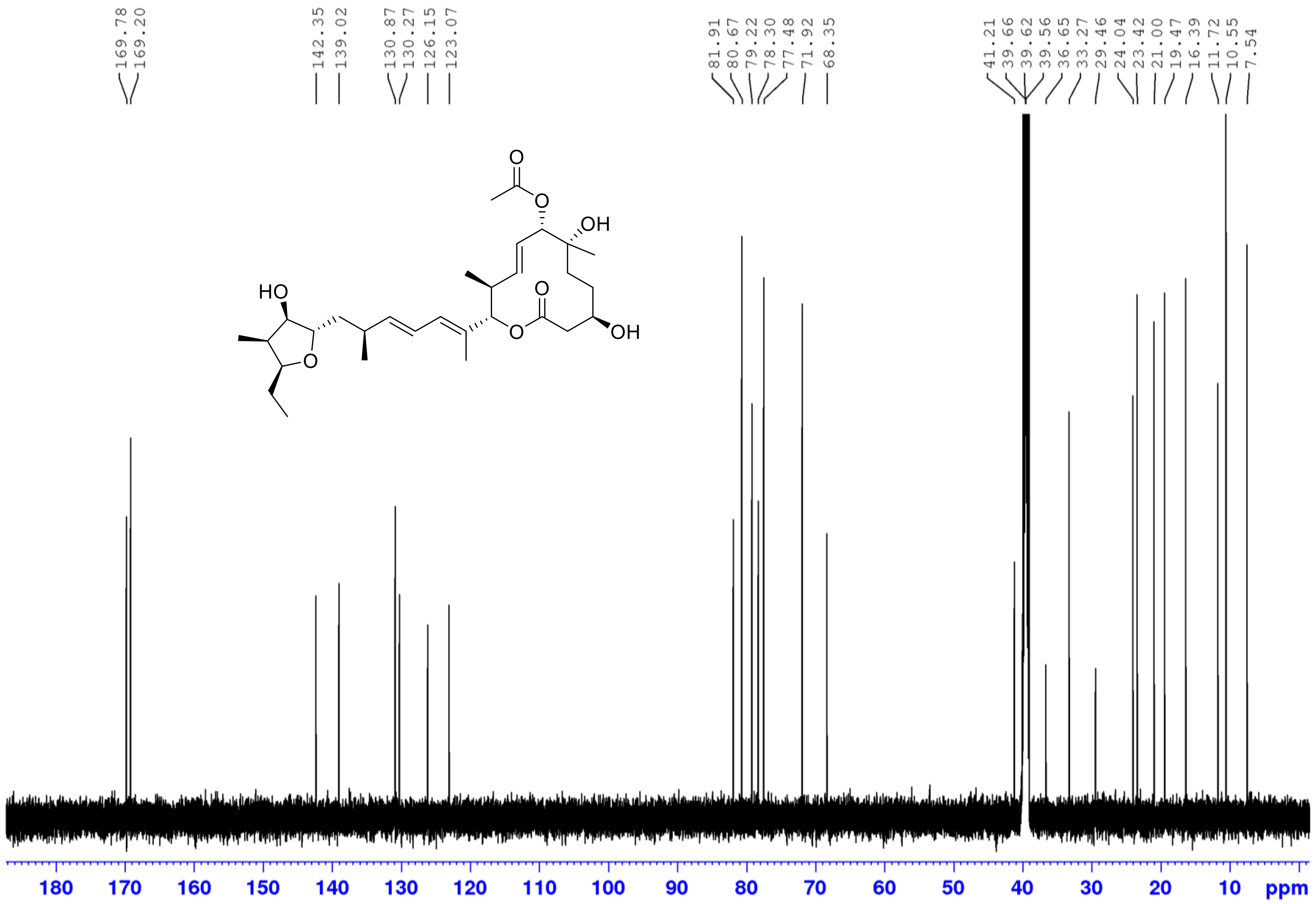

Figure S17: Inhibition of NS-1 by pladienolide B (**1**) and its congeners

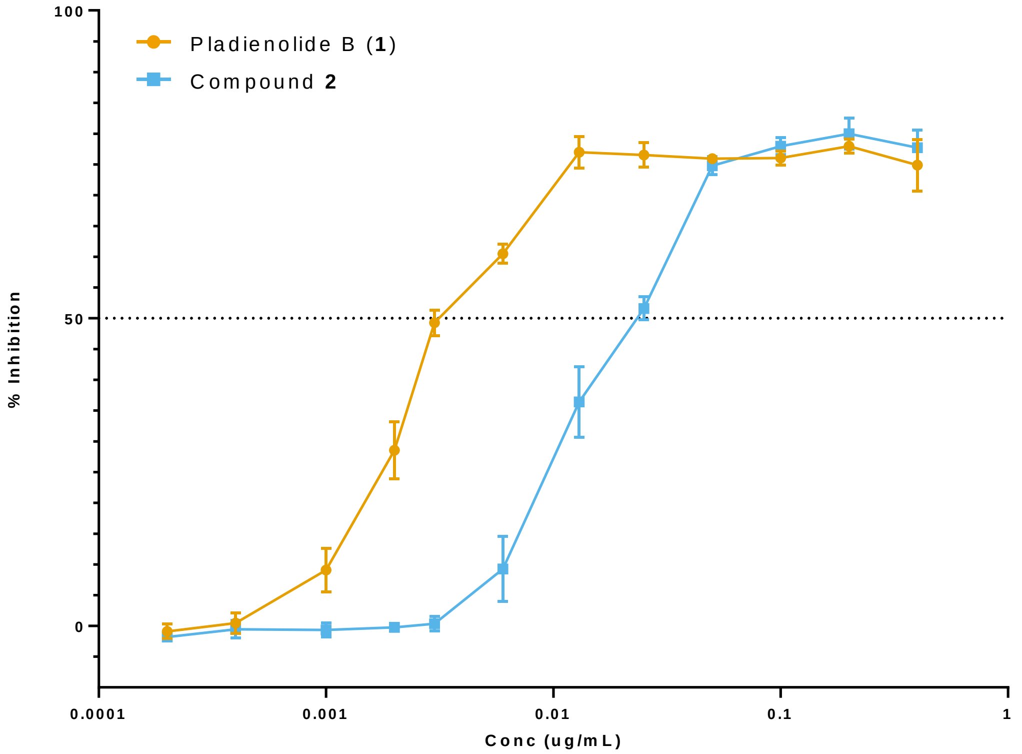

Figure S18: Inhibition of NFF by pladienolide B (**1**) and its congeners

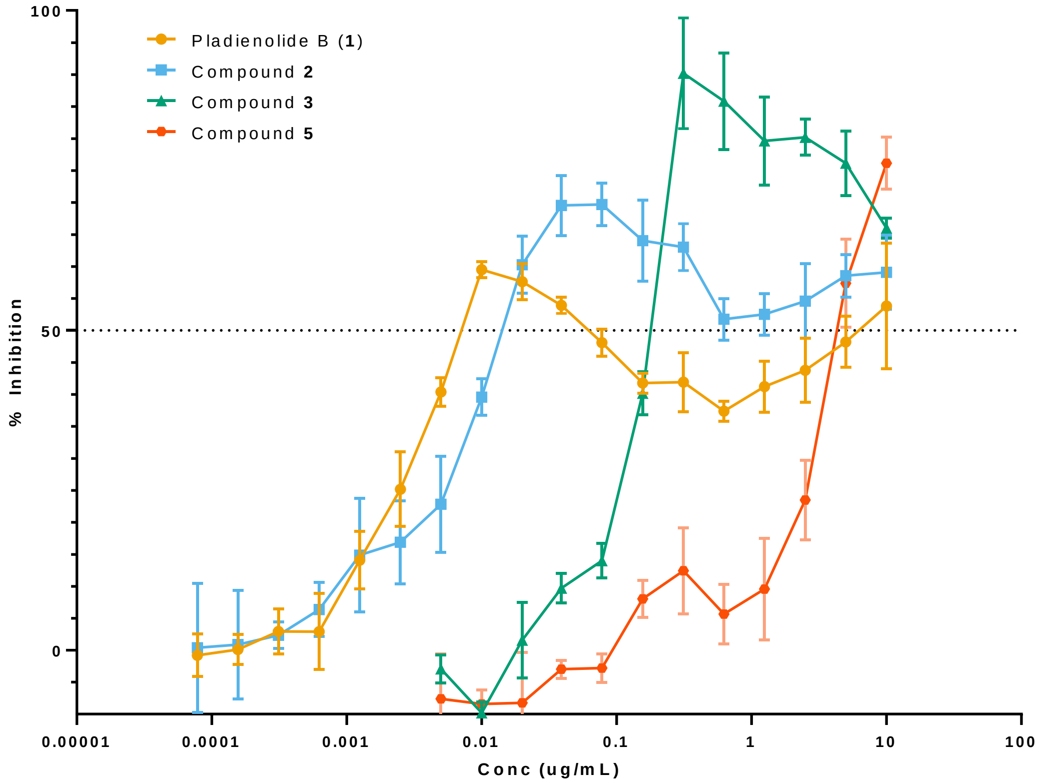

Figure S19: Inhibition of DU145 by pladienolide B (**1**) and its congeners

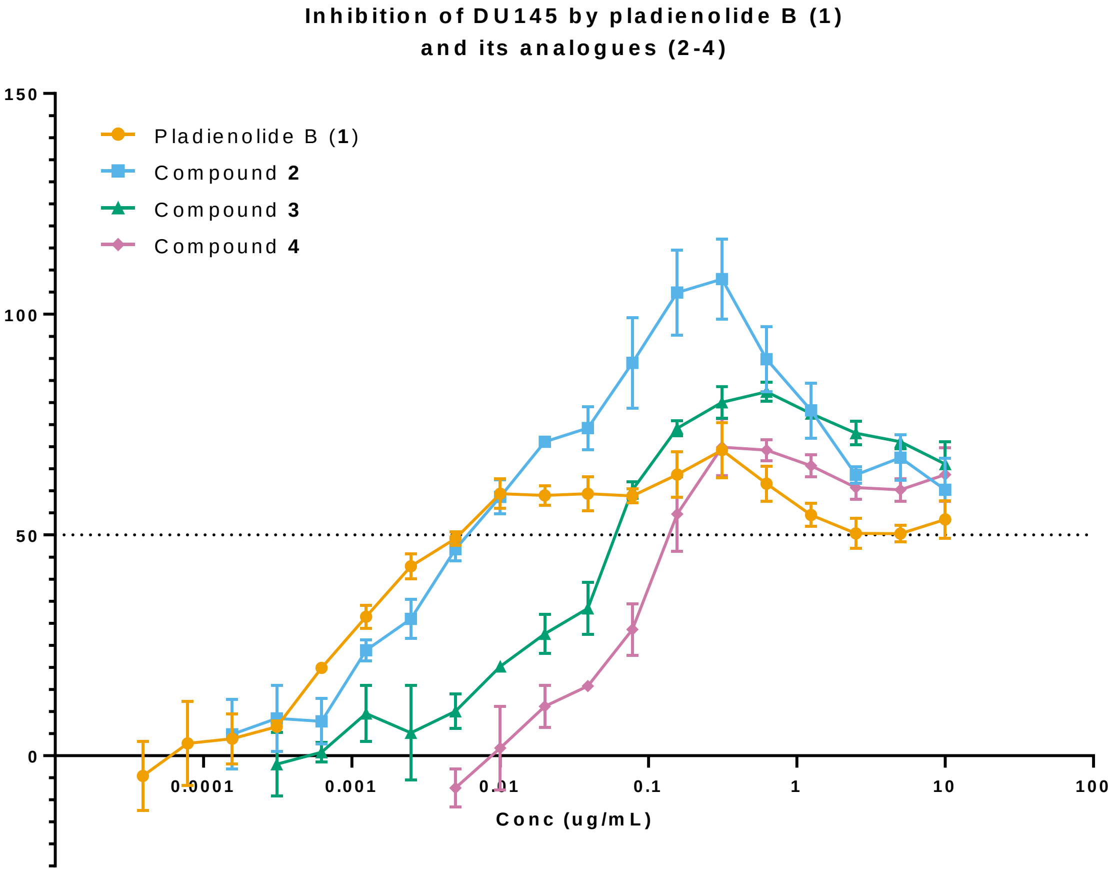

3. Supplementary Tables

Table S1: Plasmids used in this study

**Plasmid**	**Description**	**Reference**
pUZ8002	Non-transmissible oriT mobilizing plasmid	Paget et al., 1999[12]
pGP9	*Streptomyces* expression vector; propagates in *E. coli* for cloning and conjugation; Apr^R	Zhang et al., 2009[13]
pTJB1	*Streptomyces* expression vector containing *pldR* sequence cloned between *Nde*I and *Hin*dIII sites; propagates in *E. coli* for cloning and conjugation; Apr^R	This study

Table S2: Strains used in this study

**Strain**	**Description**	**Reference**
Streptomyces platensis AS6200	Pladienolide producer	This study, derivative of *Streptomyces platensis* ATCC 23948
Streptomyces platensis MA5455	Pladienolide producer	This study
Streptomyces platensis AS6242	pGP9 negative control	This study
Streptomyces platensis AS6200 (pGP9+*pldR*)	PldR overexpression strain	This study
Escherichia coli DH5a	Cloning strain. F⁻ *endA1 glnV44 thi-1 recA1 relA1 gyrA96 deoR nupG purB20* φ80d*lacZ*ΔM15 Δ(*lacZYA-argF*)U169, hsdR17($r_K^-m_K^+$), λ⁻	Hanahan, 1985[14]
Escherichia coli ET12567 (pUZ8002)	Demethylating strain for conjugation with *Streptomyces* spp. F- *dam*-13::Tn9 *dcm*-6 *hsdM hsdR zjj*-202::Tn10 *recF*143 *galK2 galT22 ara*-14 *lacY*1 *xyl*-5 *leuB6 thi*-1 *tonA*31 *rpsL*136 *his*G4 *tsx*-78 *mtl*-1 *glnV*44, pUZ8002	MacNeil, 1992[15]
Escherichia coli ET12567 (pUZ8002, pGP9)	Demethylating strain for conjugation with *Streptomyces* spp. F- *dam*-13::Tn9 *dcm*-6 *hsdM hsdR zjj*-202::Tn10 *recF*143 *galK2 galT22 ara*-14 *lacY*1 *xyl*-5 *leuB6 thi*-1 *tonA*31 *rpsL*136 *his*G4 *tsx*-78 *mtl*-1 *glnV*44, pUZ8002, pGP9	This study
Escherichia coli ET12567 (pUZ8002, pGP9+*pldR*)	Demethylating strain for conjugation with *Streptomyces* spp. F- *dam*-13::Tn9 *dcm*-6 *hsdM hsdR zjj*-202::Tn10 *recF*143 *galK2 galT22 ara*-14 *lacY*1 *xyl*-5 *leuB6 thi*-1 *tonA*31 *rpsL*136 *his*G4 *tsx*-78 *mtl*-1 *glnV*44, pUZ8002, pGP9+*pldR*	This study

Table S3: Genome Assembly Results

	**AS6200**	**MA5455**
Total Contigs	4	2
Mean Contig Length	2, 240, 097	4, 452, 871
N50	4, 651, 148	8, 608, 435
Total Bases	8, 960, 388	8, 905, 742

Table S4: Comparison of AS6200 and MA5455 contigs. Percentage pairwise identity/ percentage identical sites.

		**MA5455**	
		Contig 1 (8608435 bp)	Contig 2 (297307 bp)
**AS6200**	Contig 1 (4651148 bp)	99.5/99.7	-
	Contig 2 (3977311 bp)	99.3/99.7	-
	Contig 3 (309151 bp)	-	99.7/99.9
	Contig 4 (22778 bp)	99.7/99.8	-

Table S5: Comparison of *pld* BGC between AS6200 and Mer-1107

**Protein**	**Size (a. a.)**	**Proposed Function**	**% Similarities/ % Identities with Mer-1107 homologue**
PldAI	6543	Polyketide synthase	99/100
PldAII	2373	Polyketide synthase	95/97
PldAIII	4597	Polyketide synthase	90/91
PldAIV	3712	Polyketide synthase	100/100
PldAV	1808	Polyketide synthase	100/100
PldB	399	P450 hydroxylase	99/99
PldC	407	Acyl-transferase	100/100
PldD	462	Epoxidase	100/100
PldR	902	LuxR-like regulator	100/100

Table S6: MIC of pladienolide analogues

**Compound**	**MIC (µg ml^{-1})**					
	B. subtilis **ATCC 6633**	***S. aureus*** **ATCC 25923**	***C. albicans*** **ATCC 10231**	***S. cerevisiae*** **ATCC 9763**	**NS-1 ATCC TIB-18**	**NFF ATCC PCS-201**
**1**	>10	>10	>20	>20	0.001	0.013
**2**	>10	>10	>20	>20	0.001	0.04
**3**	>10	>10	>20	>20	0.05	0.6
**4**	>10	>10	>20	>20	0.1	0.4
**5**	>10	>10	>20	>20	0.63	10
**6**	>10	>10	>20	>20	0.08	1.25

Table S7: ^{1}H (600 MHz) and ^{13}C (150 MHz) NMR data for pladienolide B (**1**) in DMSO-d_6

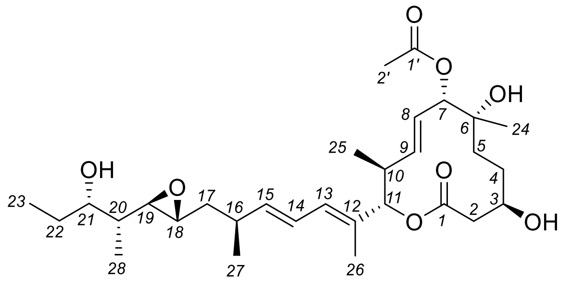

Pos.	δ_C	δ_H, mult. (*J* in Hz)	HMBC	COSY	ROESY
1	169.2				
2	39.5[a]	2.35, m	1, 3, 4	3	3, 5a, 6-OH
3	68.4	3.65, m		2, 4a/b, 3-OH	2, 4a, 5b, 3-OH
4a	29.5	1.37, m[b]	2, 3	3, 4b, 5b	3, 4b, 5b
4b		1.20, m[c]	3, 6	3, 4a, 5a	4b, 5a, 6-OH
5a	36.6	1.47, m	4, 6, 24	4b, 5b	2, 4b, 5b, 6-OH
5b		1.21, m[c]	4, 6, 7	4a, 5a	3, 4a, 5a, 7, 24
6	71.9				
7	78.3	4.89, d (9.9)	8, 9, 1'	8	5b, 24
8	126.2	5.66, dd (15.1, 9.9)	10	7, 9	9, 10
9	139.0	5.40, dd (15.1, 9.8)	7, 10, 11, 25	8, 10	8
10	39.4[a]	2.52, m	8, 9, 11, 25	9, 11, 25	8, 25, 26
11	81.9	4.86, d (10.6)	1, 9, 10, 12, 13, 26	10	13, 25
12	131.4				
13	130.0	6.01, dd (10.9, 1.1)	11, 14, 15, 26	14, 26	11, 14, 15
14	124.2	6.25, ddd (15.1, 10.9, 0.9)	13, 16	13, 15	13, 26
15	141.0	5.67, dd (15.1, 5.0)	13, 16, 17, 27	14, 16	13
16	34.7	2.41, m	14, 15, 17, 18, 27	15, 17a/b, 27	17a/b, 18, 27
17a	38.9[a]	1.58, ddd (13.7, 5.6, 5.5)	15, 16, 18, 19, 27	16, 17b, 18	16, 17b, 18, 19, 27
17b		1.36, m[b]	15, 16, 18, 27	16, 17a, 18	16, 17a, 18, 19
18	56.0	2.62, ddd (5.9, 5.6, 2.2)	17, 19, 20	17a/b	16, 17a/b, 20, 28
19	60.8	2.55, dd (8.1, 2.2)	17, 18, 20, 21, 28	20	17a/b, 20, 28
20	40.8	1.07, m	18, 19, 28	19, 28	18, 19, 22, 28
21	72.4	3.35, m	19, 23	22, 21-OH	21-OH
22	27.4	1.34, m[b]	20, 21, 23	21, 23	20, 23
23	10.5	0.82, t (7.4)	21, 22	22	22
24	24.0	1.02, s	5, 6, 7		5b, 7, 6-OH
25	16.3	0.79, d (6.8)	9, 10, 11	10	10, 11
26	11.7	1.67, d (1.1)	11, 12, 13	13	10, 14
27	20.9	1.01, d (6.7)	15, 16, 17	16	16, 17a
28	10.2	0.77, d (7.0)	19, 20, 21	20	18, 19, 20
1'	169.8				
2'	21.0	2.01, s	1'		
3-OH		4.51, d (5.4)	2, 3, 4	3	3
6-OH		4.52, s	5, 6, 7, 24		2, 4b, 5a, 24
21-OH		4.39, d (5.8)	20, 21, 22	21	21

[a] chemical shift obtained from HSQC spectrum, [b,c] overlapping resonances

Table S8: ^{1}H (600 MHz) and ^{13}C (150 MHz) NMR data for 6-deoxypladienolide (**2**) in DMSO-d_6

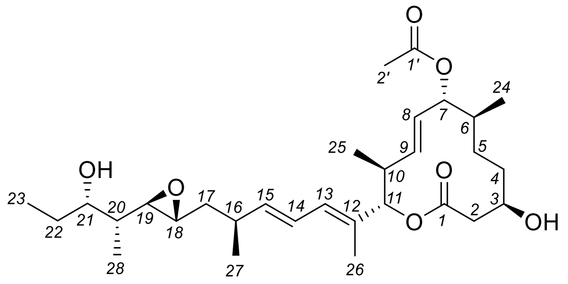

Pos.	δ_C	δ_H, mult. (*J* in Hz)	HMBC	COSY	ROESY
1	169.2				
2	41.3	2.45, m	1	2b, 3	2b, 3
		2.24, dd (13.5, 6.5)	1, 3, 4	2a, 3	2a, 3-OH
3	67.5	3.68, m		2a/b, 4b, 3-OH	2a, 3-OH
4a	29.1	1.44, m[b]		3, 4b, 5a/b	
4b		1.23, m[c]	3, 5	4a, 5a	5a
5a	28.2	1.45, m[b]		4a, 5b	4b, 5b
5b		1.18, m[c]	7	4a, 5a	5a
6	34.5	1.80, m		7, 24	
7	78.5	4.79, dd (10.1, 9.1)	6, 8, 9, 24, 1'	6, 8	8
8	129.5	5.41, dd (15.1, 9.1)	6, 9, 10, 25	7, 9	7, 10, 25
9	137.8	5.37, dd (15.1, 9.4)	7, 8, 10	8, 10	
10	39.4[a]	2.46, m	8, 11	9, 11, 25	8, 11, 25, 26
11	81.7	4.84, d (10.7)	1, 9, 10, 12, 13, 25, 26	10	10, 13, 25
12	131.4				
13	130.1	6.00, dd (10.9, 1.2)	11, 14, 15, 26	14, 26	11
14	124.2	6.25, ddd (15.1, 10.9, 0.8)	12, 13, 16	13, 15	26
15	141.0	5.66, dd (15.1, 8.1)	13, 16, 17, 27	14, 16	
16	34.7	2.41, m	14, 15, 17, 18, 19, 27	15, 17a/b, 27	27
17a	39.2[a]	1.57, ddd (13.6, 5.6, 5.4)	15, 16, 18, 19, 27	16, 17b, 18	17b
17b		1.34, m[d]	16, 18, 27	16, 17a, 18	17a
18	56.0	2.62, ddd (6.1, 5.9, 2.2)	17	17a/b	
19	60.8	2.55, dd (8.1, 2.2)	20	20	22
20	40.8	1.06, m	19, 28	19, 28	28
21	72.4	3.34, m		22, 21-OH	22
22	27.4	1.34, m[d]	20, 21, 23	21, 23	19, 21
23	10.5	0.82, t (7.4)	21, 22	22	
24	16.19	0.86, d (6.8)	5, 6, 7	6	
25	16.24	0.78, d (6.7)	9, 10, 11	10	8, 10, 11
26	11.6	1.66, d (1.0)	11, 12, 13	13	10, 14
27	20.9	1.01, d (7.0)	15, 16, 17	16	16
28	10.2	0.77, d (7.0)	19, 20, 21	20	20
1'	169.6				
2'	21.0	1.97, s	1'		
3-OH		4.56, d (5.1)	2, 3, 4	3	2b, 3
21-OH		4.39, d (5.6)	20, 21, 22	21	

[a] chemical shift obtained from HSQC spectrum, [b-d] overlapping resonances

Table S9: ^{1}H (600 MHz) and ^{13}C (150 MHz) NMR data for $\Delta^{18,19}$-pladienolide B (**3**) in DMSO-d_6

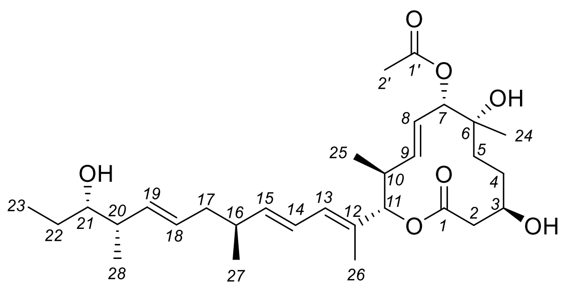

Pos.	δ_C	δ_H, mult. (*J* in Hz)	HMBC	COSY	ROESY
1	169.2				
2	39.6[a]	2.35, m	1, 3, 4	3	3, 5a
3	68.3	3.65, m		2, 4a/b, 3-OH	2, 4a, 5b
4a	29.4	1.37, m[b]	3, 5	3, 5a/b	3, 4b, 5b
4b		1.20, m[c]		3, 5a/b	4a
5a	36.6	1.46, m[d]	4, 6, 24	4a/b	2, 5b
5b		1.21, m[c]	4, 6	4a/b	3, 4a, 5a, 7
6	71.9				
7	78.3	4.89, d (9.7)	8, 9, 1'	8, 10	5b, 9, 24
8	126.2	5.66, dd (15.1, 9.7)	10	7, 9	10
9	139.0	5.40, dd (15.1, 9.9)	7, 10, 11, 25	8, 10	7
10	40.0[a]	2.50, m[a]	8, 9, 11, 25	9, 11, 25	8, 13, 25, 26
11	81.9	4.87, d (10.5)	1, 10, 13, 25, 26	10	13, 25
12	131.0				
13	130.1	6.00, dd (10.8, 1.2)	11, 14, 15, 26	14, 26	10, 11, 14, 15, 25
14	123.7	6.18, ddd (15.3, 10.8, 1.1)	13, 16	13, 15	13, 16, 26
15	141.4	5.65, dd (15.3, 7.5)	13, 16, 27	14, 16	13
16	36.5	2.24, m	14, 15, 17, 18, 27	15, 17b, 27	14
17a	39.4[a]	2.00, m	15, 16, 18, 19, 27	18	18
17b		1.95, m	15, 16, 18, 19, 27	16, 18	18
18	126.9	5.30, m[e]	17, 19, 20	17a/b	17a/b
19	135.4	5.30, m[e]	17, 18, 20, 21, 28	20	28
20	42.5	2.02, m	21, 28	19, 21, 28	21
21	74.9	3.06, m		20, 22b, 21-OH	20, 23, 28
22a	27.1	1.41, m[d]		23	22b
22b		1.17, m[a]	20, 21, 23	21, 23	22a, 23
23	10.1	0.82, t (7.4)	21, 22	22a/b	21, 22b
24	24.0	1.02, s	5, 6, 7		6-OH, 7
25	16.3	0.79, d (6.8)	9, 10, 11	10	10, 11, 13, 26
26	11.6	1.66, d (0.9)	11, 12, 13	13	10, 14, 25
27	19.6	0.95, d (6.6)	15, 16, 17	16	
28	16.6	0.90, d (6.8)	19, 20, 21	20	19, 21
1'	169.8				
2'	21.0	2.00, s	1'		
3-OH		4.51, br d		3	
6-OH		4.52, s	5, 6, 24		24
21-OH		4.25, d (5.7)	20, 21, 27	21	

[a] shift obtained from HSQC spectrum, [b-e] overlapping resonances,

Table S10: ^{1}H (600 MHz) and ^{13}C (150 MHz) NMR data for $\Delta^{18,19}$-6-deoxypladienolide B (**4**) in DMSO-d_6

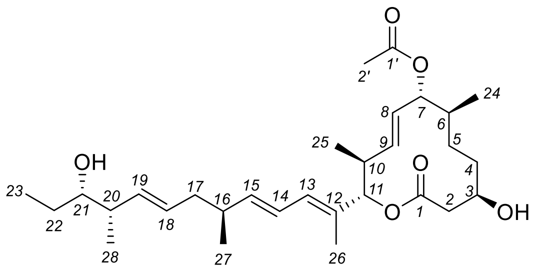

Pos.	δ_C	δ_H, mult. (*J* in Hz)	HMBC	COSY	ROESY
1	169.2				
2a	41.3	2.45, m	1, 3	2b, 3	2b, 3, 5a
2b		2.23, m[b]	1, 3, 4	2a, 3	2a, 3
3	67.5	3.68, br m		2a/b, 4a/b, 3-OH	2a/b, 4a, 5b
4a	29.1	1.44, m[c]	5	3, 5a/b	3, 4b, 5b
4b		1.23, m[d]	2, 3, 6	3, 5a/b	4a
5a	28.2	1.44, m[c]	24	4a/b	2a, 6
5b		1.18, m[d]	7	4a/b	3, 5a
6	34.5	1.80, m		7, 24	5a, 24
7	78.5	4.78, dd (10.2, 9.2)	6, 9, 24, 1'	6, 8, 10	9, 24
8	129.5	5.42, dd (15.1, 9.2)	10	7, 9	10
9	137.8	5.36, dd (15.1, 9.4)	7, 8, 10	8, 10	7, 11, 25
10	39.4[a]	2.48, m[a]	8, 9, 11, 25	9, 11, 25	8, 13, 25, 26
11	81.8	4.84, d (10.6)	1, 9, 10, 13, 25, 26	10	9, 13, 25
12	131.0				
13	130.2	6.00, br dd (10.8, 1.2)	11, 15, 26	14, 26	10, 11, 14, 15, 25
14	123.7	6.17, ddd (15.1, 10.8, 1.2)	12, 16	13, 15	13, 15, 16, 26, 27
15	141.4	5.65, dd (15.1, 5.7)	13, 16, 17, 27	14, 16	13, 14, 16, 17a, 27
16	36.5	2.23, m[b]	14, 15, 17, 18, 27	15, 17a, 27	14, 15, 17a/b, 27
17a	39.6[a]	1.99, m	16, 18, 19, 27	16, 17b, 18	15, 16
17b		1.94, m	16, 18, 19, 27	16, 17a, 18	16, 18, 27
18	126.9	5.31, m[e]	20	17a/b	17b
19	135.5	5.31, m[e]	17, 18	20	20, 21
20	42.6	2.02, m	18, 19, 21, 28	19, 21, 28	19, 28
21	75.0	3.06, m		20, 22b, 21-OH	19, 21-OH, 23, 28
22a	27.1	1.41, m[c]	20, 21, 23	23	22b
22b		1.17, m[d]	20, 21, 23	21, 23	22a
23	10.1	0.82, t (7.4)	21, 22	22a/b	21
24	16.3	0.86, d (6.7)	5, 6, 7	6	6, 7
25	16.2	0.78, d (6.7)	9, 10, 11	10	9, 10, 11, 13, 26
26	11.5	1.65, d (1.1)	11, 12, 13	13	10, 14, 25
27	19.6	0.95, d (6.7)	15, 16, 17	16	14, 15, 16, 17b
28	16.7	0.89, d (6.7)	19, 20, 21	20	20, 21
1'	169.6				
2'	21.0	1.97, (s)	1'		
3-OH		4.56, d (4.8)	3	3	
21-OH		4.24, d (5.7)	20, 21, 22	21	21

[a] shift obtained from HSQC spectrum, [b-e] overlapping resonances

Table S11: ^{1}H (600 MHz) and ^{13}C (150 MHz) NMR data for $\Delta^{18,19}$-6-deoxy-7-desacetylpladienolide B (**5**) in DMSO-d_6

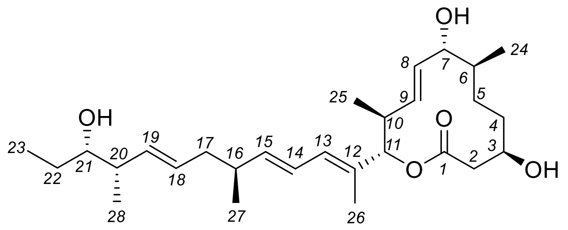

**Pos.**	**δ_C**	**δ_H, mult. (*J* in Hz)**	**HMBC**	**COSY**	**ROESY**
1	169.7				
2a	41.4	2.44, dd (13.3, 3.6)	1	2b, 3	2b, 3
2b		2.21, m[b]	1, 3, 4	2a, 3	2a, 3
3	67.7	3.65, br m		2a/b, 3-OH, 4b	2a/b, 4a/b
4a	29.6	1.41, m[c]	2, 3	5b	3
4b		1.22, m[d]	3	3	3, 7
5a	28.6	1.34, m		5b	5b
5b		1.07, m		4a, 5a	5a
6	37.1	1.45, m[c]		7, 24	
7	76.1	3.36, m	9, 24	6, 7-OH, 8	4b, 9 24
8	135.45	5.35, dd (15.0, 9.4)	10	7, 9	10
9	132.8	5.12, dd (15.0, 9.6)	7, 10, 11, 25	8, 10	7, 11, 25
10	39.44	2.46, m[a]	11	9, 11, 25	8, 25, 26
11	81.9	4.83, d (10.6)	1, 10, 12, 13, 19, 25, 26	9	9, 13, 25
12	131.2				
13	130.0	5.98, dd (10.8, 1.2)	11, 14, 26	14, 26	11, 14, 15
14	123.7	6.16, ddd (15.1, 10.8, 1.2)	12, 13, 16	13, 15	13, 16, 26, 27
15	141.3	5.64, dd (15.1, 7.6)	13, 16, 17, 27	14, 16	
16	36.5	2.24, m[b]	14, 15, 17, 27	15, 17b, 27	13, 14, 17b, 27
17a	39.41	1.99, m	16, 18, 19	18	18
17b		1.95, m	16, 18, 19	16, 18	16, 27
18	126.9	5.30, m[e]	20	17a/b, 19	17a
19	135.36	5.30, m[e]	18, 28	18, 20	28
20	42.6	2.02, m	18, 19, 21, 28	19, 21, 28	23, 28
21	75.0	3.07, m		20, 21-OH, 22b	23, 28
22a	27.1	1.41, m[c]	23	22b, 23	22b
22b		1.18, m[d]	20, 21, 23	21, 22a, 23	22a, 23
23	10.1	0.83, t (7.3)	21, 22	22a, 22b	20, 21, 22b
24	16.8	0.94, d (6.6)	5, 6, 7	6	7
25	16.5	0.80, d (7.1)	9, 10, 11	10	9, 10, 11
26	11.6	1.66, d (1.0)	11, 12, 13	13	10, 14
27	19.6	0.95, d (6.6)	15, 16, 17	16	14, 16, 17b
28	16.7	0.89, d (6.6)	19, 20, 21	20	19, 20, 21
3-OH		4.49, d (4.4)	2, 3, 4	3	
7-OH		4.55, d (4.6)	6, 7	7	
21-OH		4.25, d (5.7)	20, 21, 22	21	

[a] shift obtained from HSQC spectrum, [b-e] overlapping resonances

Table S12: ^{1}H (600 MHz) and ^{13}C (150 MHz) NMR data for isopladienolide B (**6**) in DMSO-d_6

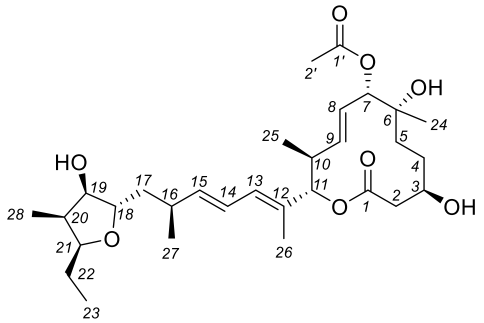

Pos.	δ_C	δ_H, mult. (*J* in Hz)	HMBC	COSY	ROESY
1	169.2				
2	39.7[a]	2.36, m	1, 3, 4	3, 3-OH	3, 3-OH, 5a
3	68.3	3.65, m		2, 4a/b	2, 3-OH, 4a, 5b
4a	29.5	1.37, m[b]		2, 3, 4b, 5b	2, 3-OH, 4b
4b		1.19, m[c]		2, 3, 4a	4b, 5a, 6-OH, 7, 24
5a	36.7	1.47, m[d]		5b, 24	2, 4b, 5b, 6-OH, 8
5b		1.21, m[c]	4, 7	4a, 5a	3, 5a, 9
6	71.9				
7	78.3	4.89, d (9.9)	8, 9, 1'	8	4b, 8, 9, 24
8	126.2	5.66, dd (15.1, 9.9)	6, 7, 10, 11	7, 9	5a, 6-OH, 7, 9, 10, 25
9	139.0	5.40, dd (15.1, 9.9)	7, 10, 11, 25	8,10	5b, 7, 8, 10, 25
10	39.6[a]	2.50, m	8, 9, 11, 12, 25	9, 11, 25	8, 9, 25, 26
11	81.9	4.87, d (10.9)	1, 10, 12, 13, 25, 26	10, 26	13, 25
12	130.9				
13	130.3	6.02, dd (10.9, 1.1)	11, 12, 14, 15, 26	14, 26	11, 14, 15, 25
14	123.1	6.20, ddd (15.2, 10.9, 1.0)	12, 13, 16	13, 15	13, 15, 16, 26, 27
15	142.4	5.73, dd (15.2, 5.7)	13, 16, 17, 27	14, 16	13, 14, 16, 17, 27
16	33.3	2.37, m	14, 15, 17, 18, 27	15, 17, 27	14, 15, 17, 27
17	41.2	1.43, m[d]	15, 16, 19, 27	16, 18	15, 16, 18, 20, 27
18	79.2	3.57, ddd (8.4, 5.2, 5.1)	16, 19, 21	17, 19	17, 27, 28
19	77.5	3.80, m	17, 21, 28	18, 19-OH, 20	17, 19-OH, 20, 28
20	39.55[a]	2.07, br m	18, 19, 22, 28	19, 21, 28	19, 21, 28
21	80.7	3.71, ddd (8.4, 5.6, 5.6)	18, 19, 23, 28	20, 22a/b	20, 22a/b, 28
22a	23.4	1.44, m[d]	18, 20, 21, 23	21, 22b, 23	21, 22b, 23
22b		1.31, m	21, 23	21, 22a, 23	21, 22a, 23
23	10.6	0.82, t (7.4)	21, 22	22a/b	22b
24	24.0	1.03, s	5, 6, 7	5a	4b, 7, 22a
25	16.4	0.795, d (6.8)	9, 10, 11	10	8, 9 ,10, 11, 13, 26
26	11.7	1.67, d (1.1)	11, 12, 13	11,13	10, 14, 25
27	19.5	0.97, d (6.6)	15, 16, 17	16	14, 15, 16, 17, 18
28	7.5	0.800, d (7.1)	19, 20, 21	20	18, 19, 19-OH, 20, 21
1'	169.8				
2'	21.0	2.01, s	1'		
3-OH		4.50, d (5.4)	2, 3, 4	3	2, 3, 4a
6-OH		4.51, s	5, 6, 7, 24		4b, 5a, 8
19-OH		4.84, d (4.8)	19	19	19, 28

[a] chemical shift obtained from HSQC spectrum, [b-d] overlapping resonances

4. References

(1) Kieser, T.; Bibb, M. J.; Buttner, M. J.; Chater, K. F.; Hopwood, D. A.; John Innes Foundation. *Practical Streptomyces Genetics*; John Innes Foundation, 2000.

(2) Eid, J.; Fehr, A.; Gray, J.; Luong, K.; Lyle, J.; Otto, G.; Peluso, P.; Rank, D.; Baybayan, P.; Bettman, B.; et al. Real-Time DNA Sequencing from Single Polymerase Molecules. *Science* **2009**, *323* (5910), 133–138.

(3) Blin, K.; Wolf, T.; Chevrette, M. G.; Lu, X.; Schwalen, C. J.; Kautsar, S. A.; Suarez Duran, H. G.; de los Santos, E. L. C.; Kim, H. U.; Nave, M.; et al. AntiSMASH 4.0—Improvements in Chemistry Prediction and Gene Cluster Boundary Identification. *Nucleic Acids Res.* **2017**, *45* (W1), W36–W41.

(4) Weber, T.; Blin, K.; Duddela, S.; Krug, D.; Kim, H. U.; Bruccoleri, R.; Lee, S. Y.; Fischbach, M. A.; Muller, R.; Wohlleben, W.; et al. AntiSMASH 3.0--a Comprehensive Resource for the Genome Mining of Biosynthetic Gene Clusters. *Nucleic Acids Res.* **2015**, *43* (W1):W237-43

(5) Altschul, S. F.; Gish, W.; Miller, W.; Myers, E. W.; Lipman, D. J. Basic Local Alignment Search Tool. *J. Mol. Biol.* **1990**, *215* (3), 403–410.

(6) Harris, R. S. Improved Pairwise Alignment of Genomic DNA, The Pennsylvania State University, 2007.

(7) Alanjary, M.; Steinke, K.; Ziemert, N. AutoMLST: An Automated Web Server for Generating Multi-Locus Species Trees Highlighting Natural Product Potential. *Nucleic Acids Res.* **2019**, *47* (W1), W276–W282.

(8) Price, M. N.; Dehal, P. S.; Arkin, A. P. FastTree 2 - Approximately Maximum-Likelihood Trees for Large Alignments. *PLoS One* **2010**, *5* (3).

(9) Gibson, D. G.; Young, L.; Chuang, R.-Y.; Venter, J. C.; Hutchison, C. A.; Smith, H. O.

Enzymatic Assembly of DNA Molecules up to Several Hundred Kilobases. *Nat. Methods* **2009**, *6* (5), 343–345.

(10) Mizui, Y.; Sakai, T.; Iwata, M.; Uenaka, T.; Okamoto, K.; Shimizu, H.; Yamori, T.; Yoshimatsu, K.; Asada, M. Pladienolides, New Substances from Culture of Streptomyces Platensis Mer-11107. III. In Vitro and in Vivo Antitumor Activities. *J. Antibiot. (Tokyo).* **2004**, *57* (3), 188–196.

(11) Komatsu, M.; Komatsu, K.; Koiwai, H.; Yamada, Y.; Kozone, I.; Izumikawa, M.; Hashimoto, J.; Takagi, M.; Omura, S.; Shin-ya, K.; et al. Engineered *Streptomyces Avermitilis* Host for Heterologous Expression of Biosynthetic Gene Cluster for Secondary Metabolites. *ACS Synth. Biol.* **2013**, *2* (7), 384–396.

(12) Paget, M. S.; Chamberlin, L.; Atrih, A.; Foster, S. J.; Buttner, M. J. Evidence That the Extracytoplasmic Function Sigma Factor SigmaE Is Required for Normal Cell Wall Structure in Streptomyces Coelicolor A3(2). *J. Bacteriol.* **1999**, *181* (1), 204–211.

(13) Zhang, C.; Ondeyka, J. G.; Zink, D. L.; Basilio, A.; Vicente, F.; Salazar, O.; Genilloud, O.; Dorso, K.; Motyl, M.; Byrne, K.; et al. Discovery of Okilactomycin and Congeners from Streptomyces Scabrisporus by Antisense Differential Sensitivity Assay Targeting Ribosomal Protein S4. *J. Antibiot. (Tokyo).* **2009**, *62* (2), 55–61.

(14) Hanahan, D. *DNA Cloning: A Practical Approach*; Glover, D. M., Ed.; IRL Press: McLean, Virginia, 1985.

(15) MacNeil, D. J.; Occi, J. L.; Gewain, K. M.; MacNeil, T. Correlation of the Avermectin Polyketide Synthase Genes to the Avermectin Structure: Implications for Designing Novel Avermectins. *Ann. N. Y. Acad. Sci.* **1994**, *721* (1 Recombinant D), 123–132.
